# Continuous(ly) missing outcome data in network meta-analysis: A one-stage pattern-mixture model approach

**DOI:** 10.1177/0962280220983544

**Published:** 2021-04

**Authors:** Loukia M Spineli, Chrysostomos Kalyvas, Katerina Papadimitropoulou

**Affiliations:** 1 Midwifery Research and Education Unit, Hannover Medical School, Hannover, Germany; 2 Biostatistics and Research Decision Sciences, MSD Europe Inc., Brussels, Belgium; 3 Clinical Epidemiology, Leiden University Medical Center, Leiden, The Netherlands; 4 Data Science and Biometrics, Danone Nutricia Research, Utrecht, The Netherlands

**Keywords:** Network meta-analysis, pattern-mixture model, continuous outcome, missing outcome data, Bayesian analysis

## Abstract

Appropriate handling of aggregate missing outcome data is necessary to minimise bias in the conclusions of systematic reviews. The two-stage pattern-mixture model has been already proposed to address aggregate missing continuous outcome data. While this approach is more proper compared with the exclusion of missing continuous outcome data and simple imputation methods, it does not offer flexible modelling of missing continuous outcome data to investigate their implications on the conclusions thoroughly. Therefore, we propose a one-stage pattern-mixture model approach under the Bayesian framework to address missing continuous outcome data in a network of interventions and gain knowledge about the missingness process in different trials and interventions. We extend the hierarchical network meta-analysis model for one aggregate continuous outcome to incorporate a missingness parameter that measures the departure from the missing at random assumption. We consider various effect size estimates for continuous data, and two informative missingness parameters, the informative missingness difference of means and the informative missingness ratio of means. We incorporate our prior belief about the missingness parameters while allowing for several possibilities of prior structures to account for the fact that the missingness process may differ in the network. The method is exemplified in two networks from published reviews comprising a different amount of missing continuous outcome data.

## 1 Introduction

**Figure 1.**
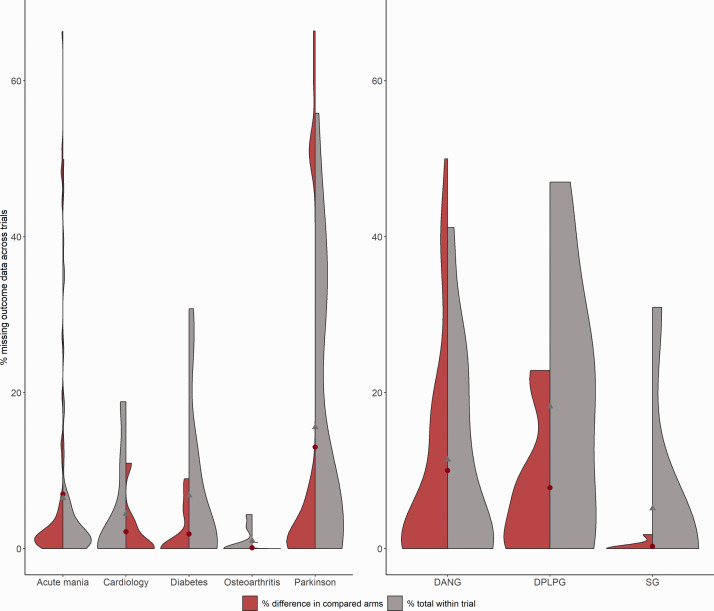
The distribution of missing continuous outcome data (MCOD; expressed as a percentage) across the different health fields in selected network meta-analyses (left plot with split violins) and the pairwise meta-analyses (right plot with split violins) from two surveys on the reporting and handling of aggregate missing outcome data.^5,6^ The red violins illustrate the density of differences in the percentage of MCOD between the compared arms across trials, and the grey violins indicate the distribution of the total percentage of MCOD across trials. The red and grey points indicate the median value in each split violin. DANG, Cochrane Depression, Anxiety and Neurosis Group; DPLPG, Cochrane Developmental, Psychosocial and Learning Problems Group; SG, Cochrane Schizophrenia Group.

Binary outcomes have drawn methodologically more attention for being the most prevalent in systematic reviews^1,2^ and rather straightforward to handle.^3^ Although less widespread in systematic reviews for being often more complex to interpret and more labour-intensive to measure compared to binary outcomes,^1^ continuous outcomes play an important role in decision-making and clinical practice. Similar to binary outcomes, continuous outcomes are also prone to missing outcome data (MOD). For instance, in a systematic review on respiratory rehabilitation in chronic obstructive pulmonary disease, Ebrahim et al.^4^ observed a MOD rate ranging from 0% to 38% across 31 included trials. In a collection of 190 Cochrane systematic reviews published between 2009 and 2012 in three mental health Cochrane Groups, 27 out of 140 selected meta-analyses considered a continuous primary outcome; of those, 14 meta-analyses reported the total number of MOD in each arm of every trial.^5^ In another collection of systematic reviews with at least three interventions published between 2009 and 2017, 92 out of 387 systematic reviews investigated a continuous primary outcome;^6^ of these, only five reported the total number of MOD in each arm of every included trial. Figure 1 illustrates the distribution of the percentage of MOD across the different health fields in both surveys.

Currently, there are a handful of methodological articles providing guidance on how to handle aggregate missing continuous outcome data (MCOD). Ebrahim et al.^4,7^ developed a three-step imputation approach to address MCOD in meta-analysis. The authors used information directly from the included trials to determine the scenarios for the imputed means and standard deviations, and they applied the same scenarios to all included trials. This approach is easy to implement; however, it fails to account for the uncertainty induced by MCOD in each arm of every trial as it merely imputes the missing observations before analysis. Depending on the amount and mechanisms of MCOD, this approach may seriously implicate the credibility of conclusions. In the case of considerable MCOD (e.g. over 20%), imputation provides spuriously more precise treatment effects^8^ and increases the risk of false-positive conclusions.

Mavridis et al.^9^ refined the two-stage pattern-mixture model, initially proposed by White et al.^10^ for binary MOD, to operate for MCOD and applied it to a published systematic review on mental health. Their approach incorporates an informative missingness parameter (IMP) that reflects researchers’ belief(s) about the missingness mechanism for each arm of every trial with the premise to adjust the within-trial results for MCOD. We distinguish this sophisticated approach for being both conceptually and statistically more appropriate to handle MCOD as it models rather than imputes the missing observations before analysis and therefore, it fully accounts for the uncertainty induced by MOD.^9^ However, the proposed two-stage approach has a shortcoming: it sets the within-trial treatment effects and standard errors fixed to the mean and variance of the missingness parameter’s distribution. Therefore, this approach does not allow the observed data to contribute to the estimation of the missingness parameter – while borrowing strength across the trials – to ‘learn’ about the missingness mechanisms.^11^ In their article, the authors study a few modelling options concerning the structure of the IMP, thus potentially overlooking alternatives that would allow the researcher to investigate in more detail the implications of different structures on the conclusions.

Occasioned by the limitations of the two-stage approach by Mavridis et al.,^9^ the present study aims to provide a one-stage pattern-mixture model approach under the Bayesian framework for MCOD to gain knowledge with respect to the missingness mechanisms across different interventions and trials in a network. A one-stage model approach allows for an eloquent synthesis of the data in a single step, and it allows us to learn about the missingness process via the estimation of the missingness parameter, thus offering an immediate advantage over the two-stage pattern-mixture model approach.

The article has the following structure. In Section 2, we introduce two published systematic reviews with network meta-analysis (NMA) with different amount of MCOD; an NMA of treatment options for Parkinson’s disease with a considerable amount of MCOD in many trials (>20% MCOD) and an NMA of physical activities for type-2 diabetes patients with a moderate amount of MCOD. In Section 3, we describe the one-stage pattern-mixture model and the missingness parameters with different structures for their prior distribution. In Section 4, we apply our method to the motivating examples. We conclude with a discussion in Section 5 and brief recommendations in Section 6.

## 2 Motivating examples

**Figure 2.**
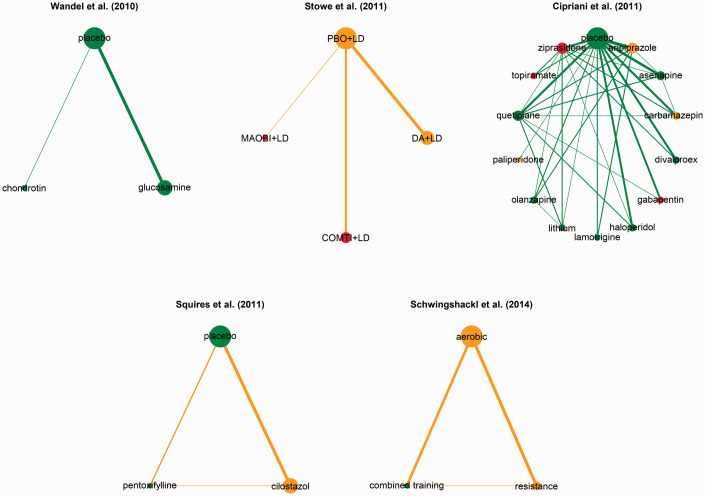
Networks on a primary continuous outcome with extractable missing continuous outcome data (MCOD) from five published systematic reviews. The size of the node is proportional to the number of observed treatment comparisons that include that node. The thickness of the edge is proportional to the number of trials that investigated that comparison. A low, moderate and large amount of percentage of total MCOD (%MCOD) is represented in each node and edge with green (%MCOD ≤5), orange and red colour (%MCOD >20). In each node, the %MCOD is the ratio of the total number of MCOD to the total number randomised across the trials that investigate the intervention. In each edge, the %MCOD is the ratio of the total number of MCOD to the total number randomised across the trials with that comparison. COMTI+LD: catechol-O-methyl transferase inhibitors plus levodopa; DA+LD: dopamine agonist plus levodopa; MAOBI+LD: monoamine oxidase type B inhibitors plus levodopa; PBO+LD: placebo plus levodopa.

We consider two motivating examples: (a) the network of Stowe et al.^12^ investigating antiparkinsonian drugs by measuring the change from baseline of patient off-time reduction (Table 1) and (b) the network of Schwingshackl et al.^13^ assessing the effect of different training modalities on HbA1c for patients with type 2 diabetes (Table 2). In both examples, negative values of mean difference (MD), and standardised mean difference (SMD) and positive values of the ratio of means (RoM) in the logarithmic scale – the most commonly used effect sizes in the synthesis of continuous outcomes – favour the first intervention in the comparison. The rationale of the choice of these networks heavily depends on the amount of missingness, quantified as the percentage of MCOD (%MCOD) from our broader collection of five networks (Figure 2; Table S1). The network of Stowe et al.^12^ has a considerable %MCOD (>20%) in many trials, whereas the network of Schwingshackl et al.^13^ suffers from a moderate amount of MCOD.

**Table 1.** Trials on four different antiparkinsonian interventions (Stowe et al.^12^)^a^

Trial	Comparisons		Placebo arm				Active arm				Total %MCOD^b^	Imbalance in %MCOD^b^
	$t_{1}$	$t_{2}$	$y_{1}^{o}$	$\sqrt{v_{1}^{o}}$	$m_{1}$	$n_{1}^{o}$	$y_{2}^{o}$	$\sqrt{v_{2}^{o}}$	$m_{2}$	$n_{2}^{o}$		
1	A	B	−0.30	0.50	7	76	−1.20	0.48	3	81	6%	5%
2	A	B	−2.47	1.13	8	12	−3.33	0.93	9	14	40%	1%
3	A	B	−0.70	0.56	2	16	−2.00	0.55	1	18	8%	6%
4	A	B	−0.77	0.49	19	46	−2.08	0.34	34	89	28%	2%
5	A	B	−0.20	0.35	0	187	−1.80	0.35	0	189	0%	0%
6	A	B	−0.90	0.50	1	100	−2.80	0.20	1	200	1%	0%
7	A	B	−0.60	0.56	4	29	−1.20	0.54	7	29	16%	−7%
8	A	B	−0.12	0.23	3	180	−1.94	0.20	6	174	2%	−2%
9	A	B	−0.30	0.50	7	76	−2.60	0.51	8	71	9%	−2%
10	A	B	−0.70	0.28	7	172	−2.40	0.26	7	174	4%	0%
11	A	B	−0.30	0.32	1	190	−2.10	0.32	1	201	1%	0%
12	A	B	−2.22	0.63	0	23	−1.74	0.49	0	23	0%	0%
13	A	B	−0.75	0.60	0	22	−1.33	0.36	4	42	6%	−9%
14	A	B	−1.22	0.51	1	53	−1.53	0.45	5	90	4%	−3%
15	A	B	−0.90	0.50	1	100	−2.50	0.25	3	201	1%	0%
16	A	B	−0.90	0.31	1	119	−2.70	0.36	118	113	34%	−50%
17	A	C	−0.90	0.40	30	74	−1.60	0.22	68	129	33%	−6%
18	A	C	−0.90	0.20	0	96	−1.00	0.14	0	174	0%	0%
19	A	C	−0.54	0.39	0	63	−0.86	0.29	0	99	0%	0%
20	A	C	−0.40	0.15	11	218	−1.20	0.15	9	218	4%	1%
21	A	C	−0.10	0.26	0	86	−1.30	0.24	0	85	0%	0%
22	A	C	−1.00	0.23	0	99	−1.50	0.26	0	98	0%	0%
23	A	C	−0.30	0.38	40	57	−1.10	0.24	88	115	43%	−2%
24	A	C	−0.67	0.37	0	58	−2.03	0.34	59	60	33%	−50%
25	A	C	−0.04	0.27	0	42	−1.61	0.28	79	40	49%	−66%
26	A	C	−0.30	0.30	0	72	−2.00	0.30	74	69	34%	−52%
27	A	C	−0.11	0.43	5	37	−1.78	0.50	81	31	56%	−60%
28	A	D	−0.40	0.15	11	218	−1.18	0.15	9	222	4%	1%
29	A	D	−0.91	0.20	0	159	−1.85	0.20	164	149	35%	−52%

A: Placebo plus levodopa; B: dopamine agonist plus levodopa; C: catechol-O-methyl transferase inhibitors plus levodopa; D: monoamine oxidase type B inhibitors plus levodopa; MCOD: missing continuous outcome data. $y_{k}^{o}$: observed mean change from baseline in arm k; $\sqrt{v_{k}^{o}}$: observed standard error in arm k; $m_{k}$: number of missing patients in arm k; $n_{k}^{o}$: the number of completers in arm k.

^a^Of the total 33 two-arm trials, we excluded three trials for reporting opposite signs in the mean change from baseline in the compared arms (log RoM cannot be calculated for these trials), and one trial due to inaccuracies in the available information regarding MCOD (Figure 1 in Stowe et al.^12^).

^b^Green indicates a low risk of attrition bias (≤5%), red indicates a substantial risk of attrition bias (>20%), and orange indicates a moderate risk of attrition bias.

**Table 2.** Trials on three different training modalities (Schwingshackl et al.^13^)

Trial^a^	Comparisons			Placebo arm				Active arm 1				Active arm 2				Total %MCOD^b^	Imbalance in %MCOD^†^
	$t_{1}$	$t_{2}$	$t_{3}$	$y_{1}^{o}$	$\sqrt{v_{1}^{o}}$	$m_{1}$	$n_{1}^{o}$	$y_{2}^{o}$	$\sqrt{v_{2}^{o}}$	$m_{2}$	$n_{2}^{o}$	$y_{3}^{o}$	$\sqrt{v_{3}^{o}}$	$m_{3}$	$n_{3}^{o}$		
1	A	B	NA	−0.4	0.10	1	19	−0.35	0.11	1	19	NA	NA	NA	NA	5%	0%
2	A	B	C	−0.12	0.10	0	72	−0.04	0.09	0	73	−0.23	0.09	0	0	0%	0%
3	A	B	C	7.42	0.43	0	12	8.24	0.61	0	12	7.53	0.30	0	0	0%	0%
4	A	B	C	−0.60	0.10	0	21	−0.20	0.05	0	23	−0.90	0.09	0	0	0%	0%
5	A	B	NA	−0.60	0.31	0	15	−0.30	0.25	0	13	NA	NA	NA	NA	0%	0%
6	A	B	NA	−0.53	0.13	1	12	−0.35	0.12	2	11	NA	NA	NA	NA	11%	−8%
7	A	B	NA	−0.30	0.16	0	30	−0.40	0.11	0	30	NA	NA	NA	NA	0%	0%
8	A	B	C	−0.43	0.12	11	49	−0.30	0.12	8	56	−0.90	0.12	6	58	13%	9%^c^
9	A	B	NA	−0.10	0.17	4	9	-0.10	0.31	4	9	NA	NA	NA	NA	31%	0%
10	A	B	C	−1.33	0.24	5	15	−0.55	0.11	5	15	−1.74	0.22	5	15	25%	0%
11	A	C	NA	6.34	0.21	0	20	6.65	0.23	0	22	NA	NA	NA	NA	0%	0%
12	A	C	NA	−0.10	0.11	0	9	−0.10	0.22	0	10	NA	NA	NA	NA	0%	0%
13	A	C	NA	6.90	0.20	1	21	7.20	0.20	0	24	NA	NA	NA	NA	2%	4%
14	A	C	NA	7.00	0.37	1	18	6.90	0.28	2	17	NA	NA	NA	NA	8%	−5%

A: aerobic; B: resistance; C: combined training; MCOD: missing continuous outcome data. $y_{k}^{o}:\;$observed mean change from baseline in arm k; $\sqrt{v_{k}^{o}}$: observed standard error in arm k; $m_{k}$: number of missing patients in arm k; $n_{k}^{o}$: the number of completers in arm k.

^a^The number of randomised participants was obtained from the corresponding published reports, and then we calculated the missing outcome data in each trial.

^b^Green implies a low risk of attrition bias (≤5%), red indicates a substantial risk of attrition bias (>20%), and orange implies a moderate risk of attrition bias.

^c^Calculated as the difference between the arms with the maximum and minimum percentage of missing outcome data.

In more detail, in the network of Stowe et al.,^12^ 10 trials (34%) have a considerable %MCOD (range: 28–56), while only six trials (21%) are MCOD-free (Table 1). Of the four treatments, placebo plus levodopa (placebo+LD) has the lowest %MCOD across trials, whereas catechol-O-methyl transferase inhibitors plus levodopa (COMTI+LD) has the largest %MCOD (%MCOD median: 35, interquartile range (IQR): 0–51). The network of Schwingshackl et al.^13^ has a moderate amount of MCOD compared to Stowe et al.^12^ as half of the trials are MCOD-free and the remaining half has moderate to considerable %MCOD (range: 8–31; Table 2). Among the three competing interventions, resistance is the most ‘problematic’ (%MCOD median: 2, IQR: 0–15) followed by aerobic (%MCOD median: 2, IQR: 0–7). Moreover, the imbalance of %MCOD within the trials is less profound in Schwingshackl et al.^13^ as the majority of trials has a lack of or low imbalance in %MCOD ( <5 in absolute value).

## 3 Methods

### 3.1 Notation

Consider a series of N trials comparing different sets of T interventions for the same patient population and health condition. In the absence of MCOD, we have information on the mean $y_{ik}\;$and the variance $v_{ik}$ of a continuous outcome (for instance, change in pain intensity scores) as measured in the $n_{ik}$ randomised participants in arm k ( $k=1,\;2,\;\ldots ,a_{i}\;\text{ with }\;a_{i}\;\text{being the number of arms in trial}\;i$) of trial i, where

$$y_{ik}∼N\left(\theta_{ik},v_{ik}\right)$$

By convention, we assume $v_{ik}$ to be known.

In the presence of MCOD, we do not have information on $y_{ik}$ and $v_{ik}$. Instead, we know the mean $y_{ik}^{o}$ (superscript to abbreviate observed) and the variance $v_{ik}^{o}$ of the continuous outcome as measured in the $n_{ik}^{o}$ participants who completed (from now on called completers) arm k of trial i out of the total randomised ( $n_{ik}$), where

$$y_{ik}^{o}∼N\left(\theta_{ik}^{o},v_{ik}^{o}\right)$$

and

$$m_{ik}∼\mathrm{Bin}\left(q_{ik},n_{ik}\right)$$

with $m_{ik}=n_{ik}-n_{ik}^{o}$ the missing participants in arm k of trial i, and $q_{ik}$ the probability of being missing. We assign a uniform prior distribution on $q_{ik}$ with support in $\left[0\text{,}1\right]$. Exclusion of MCOD corresponds to analysing the $n_{ik}^{o}$ completers in arm k of trial i. By reducing the randomised sample to the completers, exclusion of MCOD results in loss of power and may increase the risk of biased results if the reasons for premature discontinuation are informative.^10,11^

### 3.2 One-stage pattern-mixture model

In each trial, we are interested in estimating the unknown parameter $\theta_{ik}$ while accounting for the missing participants properly. For that purpose, we consider a pattern-mixture model to study the missing participants and completers jointly

(1) $$E\left(y_{ik}\right)=\theta_{ik}=\theta_{ik}^{o}\left(1-q_{ik}\right)+\theta_{ik}^{m}q_{ik}$$

where $\theta_{ik}^{m}$ is the missingness parameter that indicates the underlying mean of the continuous outcome among the missing participants in arm k of trial i. By using equation (1), the randomised sample is retained in each trial of the network to allow for inferences on the whole target population irrespective of trial completion or premature discontinuation. The available data provide no information on $\theta_{ik}^{m}$, and therefore, we have to make clinically plausible assumptions.

#### 3.2.1 Informative missingness parameters

In order to quantify the missingness process, appropriate missingness parameters have been proposed. Following Mavridis et al.,^9^ we consider the following IMPs for MCOD:

##### Informative missingness difference of means (IMDoM)

The IMDoM is defined as the difference between the mean outcome among missing participants and the mean outcome among the completers

$$\varphi_{ik}=\theta_{ik}^{m}-\theta_{ik}^{o}$$

By replacing$\theta_{ik}^{m}=\varphi_{ik}+\theta_{ik}^{o}$ in
equation (1) and rearranging, we obtain

$$\theta_{ik}^{o}=\theta_{ik}-\varphi_{ik}q_{ik}$$

The $\varphi_{ik}$s are not known and cannot be retrieved from the data, and hence we need to propose plausible values for them. Under the Bayesian framework, it naturally translates to assigning a prior distribution for $\varphi_{ik}$. A natural choice for $\varphi_{ik}$ is a normal prior distribution with mean $\Delta_{ik}$ and variance $\sigma_{ik}^{2}$ that reflect our prior belief and uncertainty about the missingness process on average, respectively, in arm k of trial i. Then, $\Delta_{ik}>0$ implies that a larger outcome on average is more likely to occur among missing participants rather than completers in arm k of trial i, $\Delta_{ik}<0$ implies the opposite, and $\Delta_{ik}=0$ indicates the missing at random (MAR) assumption on average. According to Mavridis et al.,^9^ the precision of the summary effect decreases for $\sigma_{ik}^{2}\geq 3^{2}$; afterwards, the between-trial variance becomes zero, and the increase in within-trial standard error increases further the standard error of the summary effect (Figure 5).^9^ We consider $\sigma_{ik}^{2}\geq 3^{2}$ to be *conservative*, whereas $\sigma_{ik}^{2}<1$ to be *liberal* regarding our prior belief for the missingness mechanism on average. For model identifiability, we use informative priors on $\varphi_{ik}$ following the prior distributions considered in Mavridis et al.^9^ Specifically, we use $\sigma_{ik}^{2}=1$ in all models as the primary analysis, and $\sigma_{ik}^{2}=3^{2}$ as the sensitivity analysis. With the sensitivity analysis, we aim to investigate whether and how increasing the prior variance of $\varphi_{ik}$ may impact on the investigated NMA parameters (see Section 3.4).

##### Informative missingness ratio of means (IMRoM)

The IMRoM is defined as the ratio of the mean outcome among the missing participants to the mean outcome among the completers

$$\exp\left(\omega_{ik}\right)=\frac{\theta_{ik}^{m}}{\theta_{ik}^{o}}$$

By replacing $\theta_{ik}^{m}$ in equation (1) and rearranging, we obtain

$$\theta_{ik}^{o}=\frac{\theta_{ik}}{1-q_{ik}\left(1-e^{\omega_{ik}}\right)}$$

Then, we can assign a normal prior distribution on $\omega_{ik}$ with mean $\Delta_{ik}$ and variance $\sigma_{ik}^{2}$. In line with IMDoM, we consider $\sigma_{ik}^{2}\geq 0.4^{2}$ to be *conservative*, whereas $\sigma_{ik}^{2}<0.2^{2}$ to be *liberal* regarding our prior belief for the missingness mechanism on average (Figure 5, there ^9^). We use $\sigma_{ik}^{2}=0.2^{2}$ in all models as the primary analysis, and $\sigma_{ik}^{2}=0.4^{2}$ as the sensitivity analysis to investigate the impact of increasing the prior variance of $\omega_{ik}$ on the investigated NMA parameters.

#### 3.2.2 Structural assumptions for the missingness parameters

There are various options of increasing flexibility with respect to how the IMPs across trials and arms can be structured.^11,14^ We focus on the following:

- common-within-network; the IMPs are assumed to be the same in the whole network, and only one parameter is estimated per network;
- trial-specific; the IMPs are different across trials but assumed to be the same in the compared arms resulting in down-weighting trials with unbalanced MCOD;^10^
- intervention-specific; allowing the IMP to be different across interventions but shared across trials, thus resulting in down-weighting trials with higher total MCOD.^10^

The IMPs can be further assumed to be identical, hierarchical or independent, which corresponds to hypothesising that these parameters are constant, exchangeable or different, respectively. In this work, the latter is assumed to be either uncorrelated or correlated across the arms of every trial with correlation $\mathrm{cor}\left(\varphi_{ij},\varphi_{il}\right)=\mathrm{cor}\left(\omega_{ij},\omega_{il}\right)=0.5$ for $j,l\in \left\{1,\;2,\;\ldots ,a_{i}\right\}$, and j≠l following Mavridis et al.^9^ The researchers should consider an expert opinion to define the correlation parameter,^9^ though it is not an easy task. White et al.^15^ offer a framework to elicit the correlation parameter of several arms in a trial. Furthermore, applying both correlated and uncorrelated IMPs can aid in understanding whether results are robust to the joint distribution of IMPs. Table 3 summarises all structural assumptions mentioned above for $\varphi_{ik}$ and $\omega_{ik}$ under the MAR assumption on average in the primary and sensitivity analyses. Note that in the hierarchical structure, we have assigned a uniform prior distribution on the hyper-standard deviation; other proper prior distributions may be considered as well.^16^

**Table 3.** Assumptions and prior structure of the missingness parameters $\varphi_{ik}$ and $\omega_{ik}.$

Structure	Assumption	Prior specification	
		Primary analysis	Sensitivity analysis
Identical	Common-within-network	$\varphi_{ik}=\varphi$, $\varphi ∼N\left(0,1^{2}\right)\omega_{ik}=\omega$, $\omega ∼N\left(0,0.2^{2}\right)$	$\varphi_{ik}=\varphi$, $\varphi ∼N\left(0,3^{2}\right)\omega_{ik}=\omega$, $\omega ∼N\left(0,0.4^{2}\right)$
	Trial-specific	$\varphi_{ik}=\varphi_{i}$, $\varphi_{i}∼N\left(0,1^{2}\right)\omega_{ik}=\omega_{i}$, $\omega_{i}∼N\left(0,0.2^{2}\right)$	$\varphi_{ik}=\varphi_{i}$, $\varphi_{i}∼N\left(0,3^{2}\right)\omega_{ik}=\omega_{i}$, $\omega_{i}∼N\left(0,0.4^{2}\right)$
	Intervention-specific	$\varphi_{ik}=\varphi_{t_{ik}}$, $\varphi_{t_{ik}}∼N\left(0,1^{2}\right)\omega_{ik}=\omega_{t_{ik}}$, $\omega_{t_{ik}}∼N\left(0,0.2^{2}\right)$	$\varphi_{ik}=\varphi_{t_{ik}}$, $\varphi_{t_{ik}}∼N\left(0,3^{2}\right)\omega_{ik}=\omega_{t_{ik}}$, $\omega_{t_{ik}}∼N\left(0,0.4^{2}\right)$
Hierarchical	Common-within-network	$\varphi_{ik}∼N\left(\Delta ,\sigma^{2}\right)$, $\Delta ∼N\left(0,\;1^{2}\right)$, $\sigma ∼U\left(0\text{,}1\right)\;\omega_{ik}∼N\left(\Delta ,\sigma^{2}\right)$, $\Delta ∼N\left(0,\;0.2^{2}\right)$, σ∼U(0,0.2)	$\varphi_{ik}∼N\left(\Delta ,\sigma^{2}\right)$, $\Delta ∼N\left(0,\;3^{2}\right)$, $\sigma ∼U\left(0\text{,}3\right)\omega_{ik}∼N\left(\Delta ,\sigma^{2}\right)$, $\Delta ∼N\left(0,\;0.4^{2}\right)$, σ∼U(0,0.4)
	Trial-specific	$\varphi_{ik}∼N\left(\Delta_{i},\sigma_{i}^{2}\right)$, $\Delta_{i}∼N\left(0,\;1^{2}\right)$, $\sigma_{i}∼U\left(0\text{,}1\right)\omega_{ik}∼N\left(\Delta_{i},\sigma_{i}^{2}\right)$, $\Delta_{i}∼N\left(0,\;0.2^{2}\right)$, $\sigma_{i}∼U(0\text{,}0.2)$	$\varphi_{ik}∼N\left(\Delta_{i},\sigma_{i}^{2}\right)$, $\Delta_{i}∼N\left(0,\;3^{2}\right)$, $\sigma_{i}∼U\left(0\text{,}3\right)\omega_{ik}∼N\left(\Delta_{i},\sigma_{i}^{2}\right)$, $\Delta_{i}∼N\left(0,\;0.4^{2}\right)$, $\sigma_{i}∼U(0\text{,}0.4)$
	Intervention-specific	$\varphi_{ik}∼N\left(\Delta_{t_{ik}},\sigma_{t_{ik}}^{2}\right)$, $\Delta_{t_{ik}}∼N\left(0,\;1^{2}\right)$, $\sigma_{t_{ik}}∼U\left(0\text{,}1\right)\omega_{ik}∼N\left(\Delta_{t_{ik}},\sigma_{t_{ik}}^{2}\right)$, $\Delta_{t_{ik}}∼N\left(0,\;0.2^{2}\right)$, $\sigma_{t_{ik}}∼U(0\text{,}0.2)$	$\varphi_{ik}∼N\left(\Delta_{t_{ik}},\sigma_{t_{ik}}^{2}\right)$, $\Delta_{t_{ik}}∼N\left(0,\;3^{2}\right)$, $\sigma_{t_{ik}}∼U\left(0\text{,}3\right)\omega_{ik}∼N\left(\Delta_{t_{ik}},\sigma_{t_{ik}}^{2}\right)$, $\Delta_{t_{ik}}∼N\left(0,\;0.4^{2}\right)$, $\sigma_{t_{ik}}∼U(0\text{,}0.4)$
Independent	Uncorrelated	$\varphi_{ik}∼N\left(0,1^{2}\right)\omega_{ik}∼N\left(0,0.2^{2}\right)$	$\varphi_{ik}∼N\left(0,3^{2}\right)$ $\omega_{ik}∼N\left(0,0.4^{2}\right)$
	Correlated	$\varphi_{i}∼NVN_{a_{i}}\left(\left(\begin{array}{c} 0\\ \vdots \\ 0 \end{array}\right),1^{2}\left(\begin{array}{ccc} 1 & \cdots & 1/2\\ \vdots & \ddots & \vdots \\ 1/2 & \cdots & 1 \end{array}\right)\right)\omega_{i}∼NVN_{a_{i}}\left(\left(\begin{array}{c} 0\\ \vdots \\ 0 \end{array}\right),0.2^{2}\left(\begin{array}{ccc} 1 & \cdots & 1/2\\ \vdots & \ddots & \vdots \\ 1/2 & \cdots & 1 \end{array}\right)\right)$	$\varphi_{i}∼NVN_{a_{i}}\left(\left(\begin{array}{c} 0\\ \vdots \\ 0 \end{array}\right),3^{2}\left(\begin{array}{ccc} 1 & \cdots & 1/2\\ \vdots & \ddots & \vdots \\ 1/2 & \cdots & 1 \end{array}\right)\right)\omega_{i}∼NVN_{a_{i}}\left(\left(\begin{array}{c} 0\\ \vdots \\ 0 \end{array}\right),0.4^{2}\left(\begin{array}{ccc} 1 & \cdots & 1/2\\ \vdots & \ddots & \vdots \\ 1/2 & \cdots & 1 \end{array}\right)\right)$

$NVN_{a_{i}}$: multivariate normal distribution for trial i with $a_{i}$ being the total arms; U: uniform distribution.

### 3.3 Effect measures for a continuous outcome

#### 3.3.1 Mean difference

We use an identity function to link $\theta_{ik}$ with $u_{i}$ and $\delta_{i,k1}$ in trial i as follows

$$\theta_{ik}=u_{i}+\delta_{i,k1}I\left\{k\neq 1\right\}$$

where $u_{i}=\theta_{i1}$ is the underlying mean in the baseline arm and $\delta_{i,k1}$ is the random-effect that indicates the MD between arm k and baseline arm in trial i.

#### 3.3.2 Standardised mean difference

We use the following link function

$$\theta_{ik}=u_{i}+S_{i}\delta_{i,k1}I\left\{k\neq 1\right\}$$

where $\delta_{i,k1}$ is the SMD between arm k and baseline arm in trial i, and $S_{i}$ is the pooled standard deviation

$$S_{i}=\sqrt{\frac{\sum_{k=1}^{a_{i}}v_{ik}\left(n_{ik}-1\right)}{\sum_{k=1}^{a_{i}}\left(n_{ik}-1\right)}}$$

However, in the presence of MCOD, we have no information on $v_{ik}$. Under MAR, we may assume that the pooled standard deviation among the missing participants is equal to the pooled standard deviation among the completers. Then, we can use the pooled standard deviation among the completers as the pooled standard deviation for the randomised sample. However, by doing so, we do not acknowledge our uncertainty about the MAR assumption in the estimation of the pooled standard deviation. Instead, we may assign a gamma prior distribution on the pooled variance for the randomised sample with shape and scale parameters that are defined as follows

$$S_{i}^{2}∼\Gamma \left(\frac{\sum_{k=1}^{a_{i}}\left(n_{ik}-1\right)}{2},\frac{\sum_{k=1}^{a_{i}}\left(n_{ik}-1\right)}{2\sigma_{i}^{2}}\right)$$

where $\sigma_{i}$ is the pooled standard deviation among the completers in trial i.^17^

#### Ratio of (arithmetic) means

For the RoM, the link function is the following

$$\theta_{ik}=u_{i}e^{\delta_{i,k1}I\left\{k\neq 1\right\}}$$

where $\delta_{i,k1}$ is the RoM (arm k versus baseline arm) in the logarithmic scale in trial i. This effect measure is less prevalent in systematic reviews as compared to MD and SMD;^2^ however, we have considered it for completeness.

### 3.4 Bayesian random-effects network meta-analysis model

For all the aforementioned effect measures, we assume $\delta_{i,k1}$ to follow a normal distribution with mean $\mu_{t_{ik},t_{i1}}$ and variance $\tau^{2}$ common for all observed pairwise comparisons to facilitate estimation of the parameter when there are comparisons with few trials. With $t_{ik}$, we indicate the intervention in arm k of trial i. By considering a common $\tau^{2}$, the correlation between pairs of random-effects in multi-arm trials is equal to 0.5.^18^ We apply the consistency equation, which is a linear combination of comparisons with the reference intervention of the network (here, intervention A) to obtain the treatment effects for the remaining comparisons^19^

$$\mu_{t_{ik},t_{i1}}=\mu_{t_{ik},A}-\mu_{t_{i1},A}$$

where $t_{ik}$, $t_{i1}\in \left\{B,\;C,\;\ldots ,T\right\}$.

We use the surface under the cumulative ranking curve (SUCRA) to order the interventions from the best to the worst.^20^ For the proposed one-stage pattern-mixture models, we use the normal likelihood with known variance for each arm of every trial (example 5 in the Appendix of Dias et al.^21^). For the location parameters $u_{i}$ and $\mu_{t_{i},t_{i1}}$, we consider a normal prior distribution with mean 0 and variance 10,000. We assign a half-normal prior distribution on τ with mean 0 and variance 1.^16^ Since empirical priors for $\tau^{2}$ have been proposed only for the SMD, we use this half-normal prior distribution on τ for all three effect measures. Due to many investigated models and their parameters, we consider a pragmatic approach to assess for the convergence of all model parameters. We use the Gelman–Rubin convergence diagnostic, $\hat{R}$, and we consider parameters with $\hat{R}>1.1$ to not have achieved convergence; then, the corresponding posterior distributions cannot be trusted.^22^ For parameters with a lack of convergence, we planned to look at their trace plots and autocorrelation plots to understand the cause of non-convergence. All models were implemented in JAGS via the R-package R2jags (statistical software R, version 3.6.1).^23,24^ The R-package ggplot2 was used to draw the figures in Section 4.^25^ The functions related to this manuscript are publicly available at https://github.com/LoukiaSpin/One-stage-PM-NMA-model-Continuous-Outcomes.git.

## 4 Application of the models

**Figure 3.**
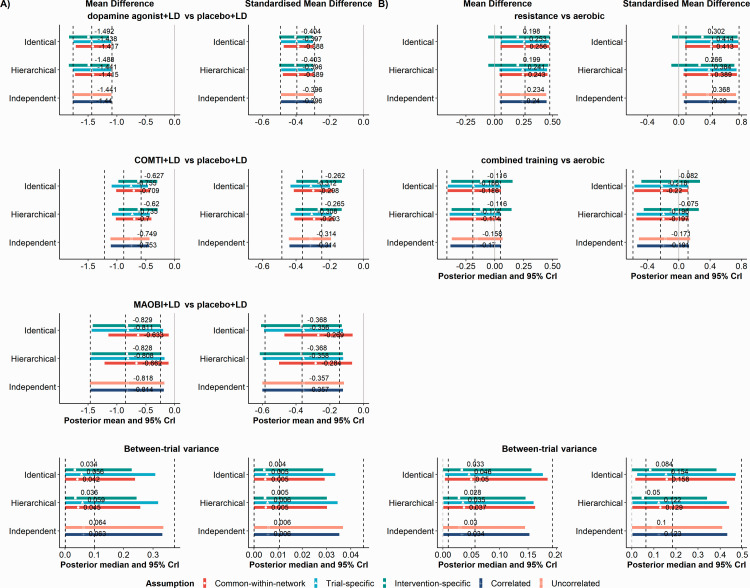
Interval plots of the posterior distribution of MD and SMD for comparisons with the reference intervention of Stowe et al.^12^ (panel A)) and Schwingshackl et al.^13^ (panel B)), and the posterior distribution of $\tau^{2}$. The one-stage pattern-mixture model under the hierarchical, identical, and independent structure of IMDoM for the assumption of common-within-network, trial-specific, intervention-specific, within-trial correlated and uncorrelated IMDoM. The vertical dashed lines refer to the posterior mean (middle line) and 95% CrI (both sides lines) under the available case analysis. COMTI+LD: catechol-O-methyl transferase inhibitors plus levodopa; CrI: credible interval; MAOBI+LD: monoamine oxidase type B inhibitors plus levodopa.

We apply the proposed models to the networks of Stowe et al.^12^ and Schwingshackl et al.^13^ We focus on MD and SMD for being the most prevalent effect measures for synthesising a continuous outcome. We present the results on log RoM for the primary and sensitivity analyses in the Supporting Information (Figure S1 –S5; Tables S2, and S4). Figure 3 depicts the posterior mean and 95% credible intervals (CrI) of all models for the comparisons with the reference intervention (i.e. placebo+LD in Stowe et al.,^12^ and aerobic in Schwingshackl et al.,^13^ respectively), and the posterior median and 95% CrI of $\tau^{2}$. In each plot, the vertical dashed lines refer to the point estimate and 95% CrI after exclusion of MCOD, which translates to the available case analysis (ACA).^26^ According to the Gelman–Rubin convergence diagnostic, convergence was achieved for all model parameters under all three effects measures and all different structural assumptions for the $\varphi_{ik}$ and $\omega_{ik}$, because $\hat{R}<1.1$ (range: 1.001 – 1.040 for $\mu_{t_{ik},A}$, $\tau^{2}$, and SUCRA values; range: 1.001 – 1.002 for $\varphi_{ik}$ and $\omega_{ik}$ under all different structural assumptions in primary and sensitivity analyses).

### 4.1 Network meta-analysis results

#### 4.1.1 Network with considerable MCOD (antiparkinsonian drugs)

The results obtained for MD and SMD analyses are very similar, following the same pattern, while on a different scale (Figure 3(A)). The posterior means for the identical and hierarchical structures are almost identical (after rounding to the second decimal) for the common-within-network, trial-specific and intervention-specific assumptions. The same conclusion can be drawn for the correlated and uncorrelated assumptions, whose estimates are found to be very similar to each other.

The intervention-specific assumption results in a slightly larger patient off-time reduction for dopamine agonist plus levodopa (DA+LD) versus placebo+LD as compared with the competing assumptions whose point estimates are identical or very similar to ACA (posterior mean of MD: −1.49 vs. −1.44). In this comparison, the results are almost interchangeable across the different assumptions because most of the included trials have low or moderate %MCOD (<10%) that is quite balanced in the compared arms as opposed to the other two comparisons.

The results for COMTI+LD versus placebo+LD are more variable across the assumptions, as expected because 6 out of 11 trials that were included suffer from considerable %MCOD that are unbalanced in the compared arms. All assumptions consistently lead to lower patient off-time reduction when compared with ACA, similarly in MD and SMD: the posterior mean is 14–30% lower than ACA across the assumptions. Among the different assumptions, the intervention-specific assumption leads to the lowest patient off-time reduction, whereas both assumptions under the independent structure yield the largest patient off-time reduction.

For the comparison of monoamine oxidase type B inhibitors plus levodopa (MAOBI+LD) versus placebo+LD, the competing assumptions yield almost interchangeable posterior means (MD ranging from −0.83 to −0.81, SMD from −0.37 to −0.36) that are close to ACA (MD: −0.83, SMD: −0.36), except for the common-within-network assumption that leads to a lower patient off-time reduction on average. Contrary to the other comparisons, the 95% CrIs are wider since two trials only inform this comparison, one of which has 35% MCOD (Table 1).

**Figure 4.**
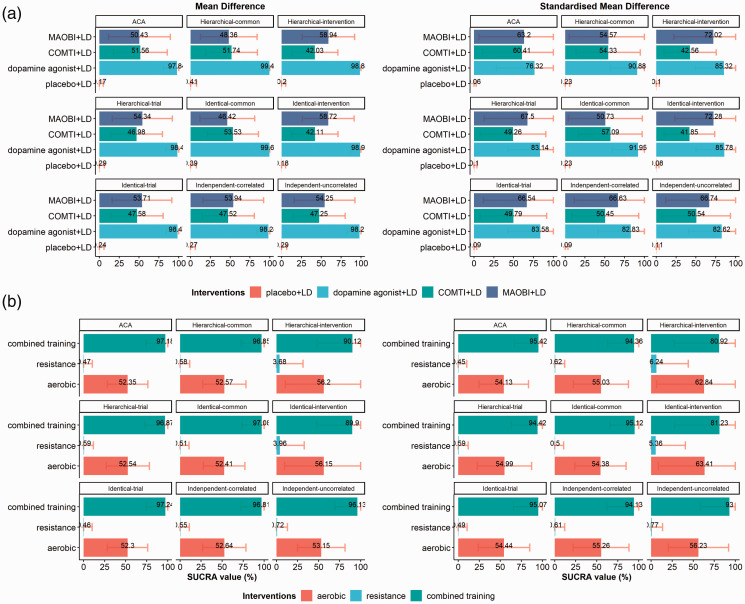
Barplots of the SUCRA values under MD, and SMD for the network of Stowe et al.^12^ (panel A)) and the network of Schwingshackl et al.^13^ (panel B)). The one-stage pattern-mixture model under the hierarchical, identical, and independent structure of IMDoM under the common-within-network, trial-specific, intervention-specific, within-trial correlated and uncorrelated assumptions. Results under ACA are also presented. ACA: available case analysis; COMTI+LD: catechol-O-methyl transferase inhibitors plus levodopa; MAOBI+LD: monoamine oxidase type B inhibitors plus levodopa; SUCRA: surface under the cumulative ranking.

All assumptions yield a lower posterior median of $\tau^{2}$ (MD: 0.03 to 0.06, SMD: 0.004 to 0.006) with narrower 95% CrI compared with ACA (MD: 0.1, SMD: 0.01) which indicates that all assumptions have explained a substantial part of the between-trial variance (MD: 40% to 70%, SMD: 40% to 60% compared to ACA). The intervention-specific assumption provides the lowest posterior median and narrowest 95% CrI of $\tau^{2}$, followed by the common-within-network assumption. The posterior median of $\tau^{2}$ is similar in the trial-specific assumption and the independent structure. According to SUCRA values, placebo+LD and DA+LD are consistently the worst and the best interventions, respectively (Figure 4(a)). The hierarchy is uncertain for COMTI+LD and MAOBI+LD due to overlapping 95% CrI across all models, which is attributed to considerable %MCOD in the corresponding trials.

By increasing the variance of the prior distribution for IMDoM at 3^2^, and therefore our uncertainty on our belief about the MAR assumption, the results follow the same pattern with the primary analysis (only results on MD are shown), but the 95% CrIs are now wider overall (Figure S4A in the Supporting Information).

#### 4.1.2 Network with moderate MCOD (different training modalities)

In line with the previous example, MD and SMD provide very similar results in terms of pattern, but on a different scale (Figure 3(B)). The point estimates are almost identical under the hierarchical and identical structures for the common-within-network, trial-specific and intervention-specific assumptions, and similar for the correlated and uncorrelated assumptions under the independent structure. The intervention-specific assumption leads to a slightly large reduction in HbA1c in favour of resistance, but the evidence is weak (the 95% Crl includes zero). All other assumptions give similar results with each other and with ACA strongly favouring resistance. Furthermore, under the intervention-specific assumption, the comparison of combined training versus aerobic results in lowering the reduction in HbA1c by approximately 37% compared with the competing assumptions. In contrast, all other assumptions result in similar findings with each other and with ACA but slightly more precise than ACA. Except for two trials with considerable %MCOD, the low to moderate %MCOD across and within the trials may explain the low variability of the results across the assumptions for each comparison with aerobic.

All assumptions lead to a lower posterior median and wider 95% CrI of $\tau^{2}$ (MD: 0.03–0.05, SMD: 0.05–0.16) as compared to ACA (MD: 0.06, SMD: 0.18). Identical structure yields a larger posterior median and wider 95% CrI of $\tau^{2}$ for each assumption as compared to the hierarchical structure. In both MD and SMD, the intervention-specific assumption under the hierarchical structure yields the lowest posterior median and most precise 95% CrI of $\tau^{2}$.

According to SUCRA values, combined training and resistance are consistently the best and the worst interventions across all assumptions, respectively, except for the intervention-specific assumption that leads to overlapping 95% CrIs (Figure 4(b)). The intervention-specific assumption yields wider CrI for comparisons with aerobic, which can explain the wide 95% CrI for SUCRA as well.

In line with the previous network, increasing the prior variance of the prior distribution of IMDoM at 3^2^ leads to results of the same pattern with the primary analysis (only results on MD are shown), but with less precise estimates overall (Figure S4A in the Supporting Information).

### 4.2 Learning about the missingness mechanism

**Figure 5.**
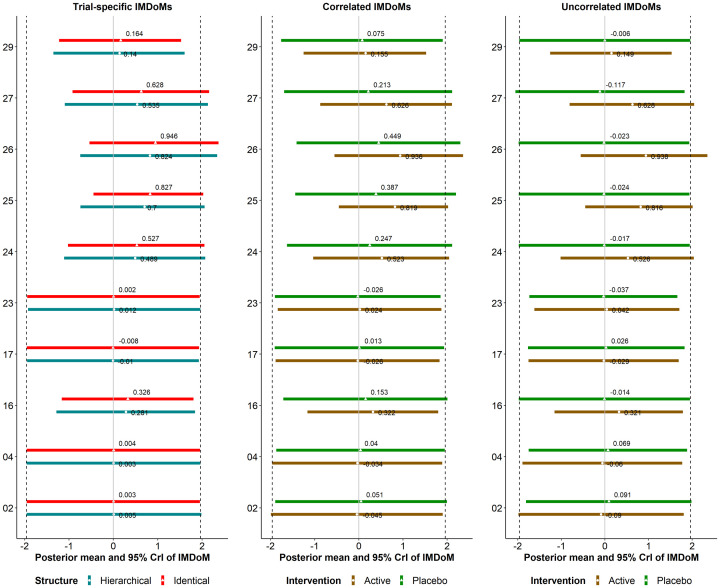
Interval plots of the posterior distribution of IMDoM using MD in the network of Stowe et al.^12^ The one-stage pattern-mixture model under the hierarchical and identical structure assuming trial-specific IMDoMs and under the independent structure assuming within-trial correlated and uncorrelated IMDoMs. The vertical lines indicate the prior distribution for IMDoM. Only the trials with considerable participant losses are presented. COMTI+LD: catechol-O-methyl transferase inhibitors plus levodopa; CrI: credible interval; DA+LD: dopamine agonist plus levodopa; IMDoM; informative missingness difference of means; MAOBI+LD: monoamine oxidase type B inhibitors plus levodopa; PBO+LD: placebo plus levodopa.

The posterior distribution of IMDoM under the common-within-network and intervention-specific assumptions is given in Table 4 for both network examples. A posterior mean away from zero is an indication that the MAR assumption may not be plausible; that is, the missingness process may be informative. Similarly, a 95% CrI excluding zero and protruding from the interval of the prior distribution of IMDoM is a strong indication of informative missingness; otherwise, the data provide little information to conclude for or against the MAR assumption. For the remaining structural assumptions of IMDoM, the posterior distribution for the most ‘problematic’ studies (i.e. studies with a considerable amount of MCOD) is shown in Figure 5 for the network of Stowe et al.^12^ The vertical lines in each plot refer to the prior mean (middle grey line) and 95% prior interval (both sides dashed lines) under the MAR assumption on average.

**Table 4.** Posterior mean and 95% CrI of IMDoM under different structural assumptions.^a^

Network	Interventions	Assumptions about the structure of IMDoM ( $\varphi_{ik}$)			
		Common-within-network		Intervention-specific	
		Hierarchical $\varphi_{ik}∼N\left(\Delta ,\sigma^{2}\right)$, $\Delta \;∼N\left(0,\;1^{2}\right)$, σ ∼U(0,1)	Identical $\varphi_{ik}=\varphi$, $\varphi ∼N\left(0,1^{2}\right)$	Hierarchical $\varphi_{ik}∼N\left(\Delta_{t_{ik}},\sigma_{t_{ik}}^{2}\right)$, $\Delta_{t_{ik}}∼N\left(0,\;1^{2}\right)$, $\sigma_{t_{ik}}∼U(0\text{,}1)$	Identical $\varphi_{ik}=\varphi_{t_{ik}}$, $\varphi_{t_{ik}}∼N\left(0,1^{2}\right)$
Antiparkinsonian drugs^12^	COMTI+LD	0.99 (0.11, 1.82)	1.02 (0.23, 1.80)	1.25 (0.22, 2.22)	1.29 (0.34, 2.20)
	DA+LD			0.02 (−1.49, 1.49)	0.05 (−1.38, 1.48)
	MAOBI +LD			0.12 (−1.36, 1.58)	0.15 (−1.23, 1.51)
	PBO+LD			0.56 (−0.91, 1.97)	0.61 (−0.79, 1.99)
Training modalities^13^	Aerobic	−0.13 (−1.94, 1.72)	−0.11 (−2.04, 1.80)	−0.18 (−1.62, 1.29)	−0.24 (−1.67, 1.25)
	Combined training			0.74 (−0.97, 2.42)	0.84 (−0.88, 2.48)
	Resistance			−0.61 (−2.17, 0.97)	−0.71 (−2.28, 0.91)

COMTI+LD: catechol-O-methyl transferase inhibitors plus levodopa; CrI: credible interval; DA+LD: dopamine agonist plus levodopa; IMDoM: informative missingness difference of means; MAOBI+LD: monoamine oxidase type B inhibitors plus levodopa; PBO+LD: placebo plus levodopa.

^a^Mean difference with informative missingness difference of means.

#### 4.2.1 Network with considerable MCOD (antiparkinsonian drugs)

The results under the common-within-network assumption suggest likely informative missingness as point estimates are positive (hierarchical: 0.99, identical: 1.02) and their corresponding CrIs do not include zero. However, the 95% CrIs do not protrude the upper bound of the prior interval (Table 4). The same conclusions are drawn when we assume a larger prior variance of IMDoM; though the posterior mean is larger (hierarchical: 1.17, identical: 1.20) and the 95% CrI are wider when compared to the primary analysis, as expected (Table S3). However, the common-within-network assumption does not reveal the source(s) of such informative missingness. On the contrary, the intervention-specific assumption indicates that for all interventions, except COMTI+LD, the data provide little information to conclude in favour of or against the MAR assumption as the 95% CrIs of the corresponding posterior distributions include zero (Table 4). Only the posterior mean and 95% CrI for COMTI+LD are far from zero suggesting informative missingness. The conclusions do not change when we assume a larger prior variance of IMDoM (Table S3). This is not surprising because COMTI+LD has attracted the highest %MCOD in the network (Table 1). COMTI+LD may also be responsible for pulling the posterior distribution of IMDoM away from zero under the common-within-network assumption. Conclusions are the same in both structures, though the identical structure provides more precise results than the hierarchical structure.

The trial-specific assumption further indicates that in trials with total %MCOD above 20% and severe imbalance in the compared arms (i.e. the trials that compare COMTI+LD with placebo+LD; Table 1), missing participants may have a smaller patient off-time reduction on average than completers, though the indication is weak (Figure 5). For these trials, the assumption of within-trial correlated IMDoMs provides further insights on the missingness process as it reveals that this behaviour is more profound in the active arm (i.e. COMTI+LD) than in the placebo arm (Figure 5). However, the assumption of within-trial uncorrelated IMDoMs indicates that only missing participants receiving COMTI+LD in these trials appear to have a smaller patient off-time reduction on average than completers (Figure 5). It is evident that by using different assumptions about the missingness parameter, we gain a different level of knowledge regarding the missingness mechanisms in the network.

#### 4.2.2 Network with moderate MCOD (different training modalities)

**Figure 6.**
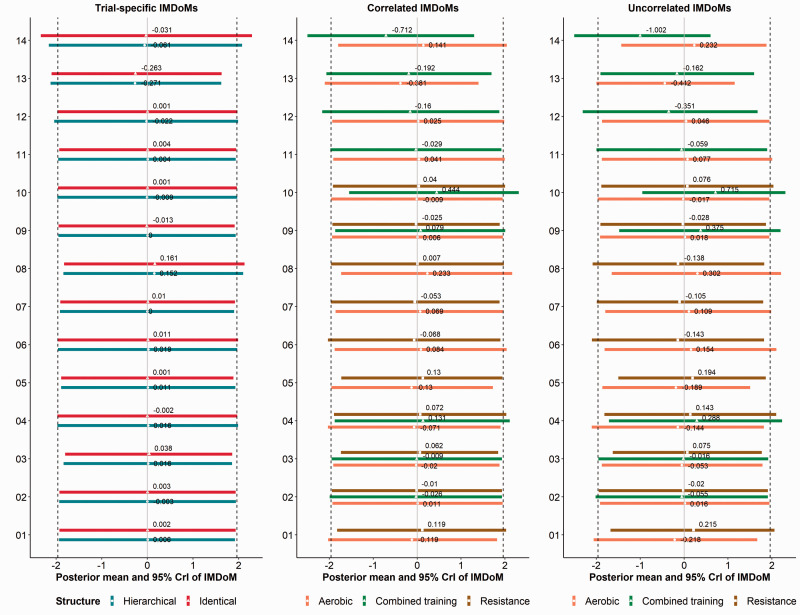
Interval plots of the posterior distribution of IMDoM using MD in the network of Schwingshackl et al.^13^ The one-stage pattern-mixture model under the hierarchical and identical structure assuming trial-specific IMDoMs and under the independent structure assuming within-trial correlated and uncorrelated IMDoMs. The vertical lines indicate the prior distribution for IMDoM. CrI: credible interval; IMDoM: informative missingness difference of means.

The data provide little information to conclude for or against the MAR assumption using the common-within-network assumption given the wide CrIs of both hierarchical and identical structures that include zero (Table 4). This finding is shared in the results under the intervention-specific and trial-specific assumptions where for all treatments and trials, the intervals of the posterior distribution of IMDoM are considerably wide and include zero (last three lines of Table 4; Figure 6). The conclusions are similar for both assumptions under the independent structure (Figure 6). Contrary to the network of antiparkinsonian drugs, the trials of this network suffer mildly from MCOD, which are balanced in the compared arms. Therefore, there is not enough information to conclude for or against the MAR assumption using different assumptions for the missingness parameter.

## 5 Discussion

We have proposed a one-stage pattern-mixture model approach under the Bayesian framework that accounts for MCOD from all trials in a single step while allowing the observed data to contribute to the estimation of the missingness parameters to learn about the missingness mechanism. The hierarchical structure of the proposed models facilitates the incorporation of various prior structures and assumptions about the missingness parameter to investigate the implications of MCOD on the conclusions. These features make the proposed model approach particularly attractive to handle aggregate MCOD properly. On the contrary, the two-stage approach does not offer enough flexibility in the analysis of MCOD as it requires strong assumptions about the estimated within-trial variance (considered known) and the missingness parameter (considered independent of the amount of MOD).^9^

Due to variation in the amount of MCOD within and across the trials, especially, in the network of Stowe et al.^12^ (Table 1), we consider the independent structure for the missingness parameters to be the most plausible, and the common-within-network assumption to be the least plausible in our motivating examples. The intervention-specific assumption is particularly relevant if one is interested to learn about the missingness mechanism in each or specific intervention(s). Common-within-network is a strong and perhaps the least realistic assumption. In a typical network with different trial-designs (e.g. placebo-controlled and active-controlled) and different interventions (e.g. placebo, active and old interventions), the amount of and reasons for MOD may vary across all arms and trials making the common-within-network assumption implausible. The trial-specific assumption is plausible when trials with different characteristics (in terms of design and conduct) are associated with different mechanisms of MOD.^27–29^ For further discussion on the situations to consider the different prior structures and assumptions for the missingness parameter (Table 3), the interested readers can refer to the literature.^11,14,29^

There is already a simulation study on the comparison of different one-stage pattern-mixture models for aggregate binary MOD.^29^ This study considered the identical and hierarchical structures for the common-within-network, intervention-specific and trial-specific assumptions for the missingness parameter. In the present study on continuous outcomes, we observed the same behaviour of the proposed methods with the one from the simulation study. For instance, the intervention-specific prior structure led to larger posterior standard deviation of the treatment effects (similarly for the hierarchical and identical assumption) as compared to the other structures, especially, for a large amount of MCOD in the network (Figure 3). We expect the one-stage pattern-mixture model for continuous MOD to behave similarly to the one-stage pattern-mixture model for binary MOD for the different structural assumptions of the missingness parameter.

Furthermore, modelling MCOD appeared to have explained part of the between-trial variance in both networks, and particularly, under the intervention-specific assumption which yielded the smallest $\tau^{2}$. The benefits of modelling rather than excluding (or imputing) MOD in the estimation of $\tau^{2}$ have been already mentioned.^9,14,30^ Modelling MOD offers a trade-off between inflation in the within-trial variance and reduction in $\tau^{2}$ which can be considerable when the amount of MOD is substantial. The opposite holds when MODs are excluded or imputed.

We illustrated the proposed one-stage pattern-mixture model approach using three different effect measures for the continuous outcome. In line with Mavridis et al.,^9^ we apply MD and SMD in conjunction with the IMDoM, and log RoM together with the log IMRoM, because they are intuitively related. To select among these three effect measures for a specific outcome, the researcher should consider the trade-off among the ease of interpretation, statistical properties (e.g. low variability of the treatment effect across the trials^31^) and the goodness of fit. According to the posterior mean of residual deviance, we found that using the log RoM, none of the models fit the data adequately for the network of Stowe et al.^12^ (Table S5 in the Supporting Information). In the network of Cipriani et al.,^32^ none of the models fit the data adequately for MD and SMD (Table S5 in the Supporting Information). For a discussion on the statistical properties and performance of these effect measures in the synthesis of trials, the readers should refer to the relevant literature.^33–35^

The proposed one-stage pattern-mixture model approach can be particularly useful in living systematic reviews,^36^ where learning about the missingness process via the estimated missingness parameters can inform the design of a future randomised trial. For instance, assuming that Stowe et al.^12^ was a living systematic review, we have learned that trials comparing COMTI+LD with placebo+LD have the most participant losses (Table 1) and missing participants randomised in COMTI+LD tend to have smaller patient off-time reduction as compared to completers in that arm (according to the intervention-specific assumption and the independent structure). Moreover, scrutiny of the COMTI+LD versus placebo+LD trials in the network using the Cochrane Risk of Bias tool will further shed light on why participant losses are almost negligible in some trials ( <4%) but considerable in others ( <30%). All this information will allow the trialists of a future COMTI+LD versus placebo+LD trial to undertake proactive plans to increase retention of the participants in the COMTI+LD arm.^37^

We were able to extract MOD in each arm of every trial in five networks only (out of the 92 networks with a continuous primary outcome). Therefore, an empirical study using this dataset would not offer sufficient evidence to compare the one-stage with the two-stage pattern-mixture model empirically. It is particularly challenging to collect a sufficient number of networks (or meta-analyses) to conduct an empirical study concerning MOD as the ability to extract MOD, and the quality of the extraction strongly depends on the reporting quality of the systematic reviews under investigation.^38^ However, a recent simulation study investigated the performance of modelling the exact distribution (one-stage approach) versus the approximately normal distribution (two-stage approach) of aggregate binary data in the presence of MOD in a triangle of two-arm trials (submitted). Both approaches showed substantial bias overall in the relative treatment effects, especially, for comparisons with the non-reference interventions of the network, when the amount of MOD was considerable. However, the one-stage approach is both conceptually and statistically more appropriate than the two-stage approach when the approximate normality assumption cannot be defended; for instance, the outcome is skewed and/or trials have small size.^39^ Since the proposed one-stage pattern-mixture model approach is based on the normal distribution (as the exact likelihood), we would expect some bias in the treatment effects when the synthesis dataset is dominated by small trials with a skewed outcome. In these situations, we recommend that until a more competent one-stage model is developed, the researchers apply our proposed one-stage PM approach to handle MCOD, and they fully acknowledge the limitations of this approach when they discuss the results.

## 6 Conclusions

Similar to binary MOD, a proper analysis of MCOD should be discussed thoroughly already in the protocol phase of the systematic review. The analysis plan should comprise the description of the one-stage model with respect to the proper assumptions and prior structure of the missingness parameter. For this purpose, prior knowledge (or expectations) of the missingness process that aligns with the interventions and the design of the trials on the condition of interest is necessary. For instance, inpatients randomised to placebo are more likely to leave the trial early for not experiencing immediate improvement in their schizophrenia symptoms as compared to participants randomised to antipsychotics.^27^ In the absence of such knowledge, we recommend that the researchers consider the intervention-specific assumption alongside the hierarchical structure under the MAR assumption (with liberal uncertainty about that belief) in the primary analysis and investigate the robustness of the results under the independent structure while increasing the variance of the prior distribution of the missingness parameter, as a sensitivity analysis. In a network with few comparisons, which are informed by a handful of trials, the identical structure may be advantageous to the independent structure for estimating comparatively fewer missingness parameters. Then, the hierarchical structure alongside the intervention-specific assumption may be considered as a sensitivity analysis. The proposed models can also be applied in a pairwise meta-analysis straightforward to benefit from the variety of structural assumptions about the missingness parameter.

## Supplemental Material

sj-pdf-1-smm-10.1177_0962280220983544 - Supplemental material for Continuous(ly) missing outcome data in network meta-analysis: A one-stage pattern-mixture model approach Click here for additional data file. Supplemental material, sj-pdf-1-smm-10.1177_0962280220983544 for Continuous(ly) missing outcome data in network meta-analysis: A one-stage pattern-mixture model approach by Loukia M Spineli, Chrysostomos Kalyvas and Katerina Papadimitropoulou in Statistical Methods in Medical Research

## References

[1] Davey J Turner RM Clarke MJ, et al. Characteristics of meta-analyses and their component studies in the Cochrane Database of Systematic Reviews: a cross-sectional, descriptive analysis. BMC Med Res Methodol 2011; 11: 160. 22114982 10.1186/1471-2288-11-160 PMC3247075

[2] Nikolakopoulou A Chaimani A Veroniki AA, et al. Characteristics of networks of interventions: A description of a database of 186 published networks. PLoS One 2014; 9: e86754. 24466222 10.1371/journal.pone.0086754 PMC3899297

[3] Akl EA Kahale LA Agoritsas T, et al. Handling trial participants with missing outcome data when conducting a meta-analysis: a systematic survey of proposed approaches. Syst Rev 2015; 4: 98. 26202162 10.1186/s13643-015-0083-6 PMC4511978

[4] Ebrahim S Johnston BC Akl EA, et al. Addressing continuous data measured with different instruments for participants excluded from trial analysis: a guide for systematic reviewers. J Clin Epidemiol 2014; 67: 560–570. 24613497 10.1016/j.jclinepi.2013.11.014

[5] Spineli LM Pandis N Salanti G. Reporting and handling missing outcome data in mental health: a systematic review of Cochrane systematic reviews and meta-analyses. Res Synth Meth 2015; 6: 175–187. 10.1002/jrsm.1131 26099485

[6] Spineli LM Yepes-Nuñez JJ Schünemann HJ. A systematic survey shows that reporting and handling of missing outcome data in networks of interventions is poor. BMC Med Res Methodol 2018; 18: 115. 30355280 10.1186/s12874-018-0576-9 PMC6201503

[7] Ebrahim S Akl EA Mustafa RA, et al. Addressing continuous data for participants excluded from trial analysis: a guide for systematic reviewers. J Clin Epidemiol 2013; 66: 1014–1021.e1. 23774111 10.1016/j.jclinepi.2013.03.014

[8] Spineli LM Kalyvas C. Comparison of exclusion, imputation and modelling of missing binary outcome data in frequentist network meta-analysis. BMC Med Res Methodol 2020; 20: 48. 32111167 10.1186/s12874-020-00929-9 PMC7049189

[9] Mavridis D White IR Higgins JP, et al. Allowing for uncertainty due to missing continuous outcome data in pairwise and network meta-analysis. Stat Med 2015; 34: 721–741. 25393541 10.1002/sim.6365 PMC6585809

[10] White IR Higgins JP Wood AM. Allowing for uncertainty due to missing data in meta-analysis–part 1: two-stage methods. Stat Med 2008; 27: 711–727. 17703496 10.1002/sim.3008

[11] Turner NL Dias S Ades AE, et al. A Bayesian framework to account for uncertainty due to missing binary outcome data in pairwise meta-analysis. Stat Med 2015; 34: 2062–2080. 25809313 10.1002/sim.6475 PMC5054891

[12] Stowe R Ives N Clarke CE, et al. Meta-analysis of the comparative efficacy and safety of adjuvant treatment to levodopa in later Parkinson's disease. Mov Disord 2011; 26: 587–598. 21370258 10.1002/mds.23517

[13] Schwingshackl L Missbach B Dias S, et al. Impact of different training modalities on glycaemic control and blood lipids in patients with type 2 diabetes: a systematic review and network meta-analysis. Diabetologia 2014; 57: 1789–1797. 24996616 10.1007/s00125-014-3303-z

[14] Spineli LM. An empirical comparison of Bayesian modelling strategies for missing binary outcome data in network meta-analysis. BMC Med Res Methodol 2019; 19: 86. 31018836 10.1186/s12874-019-0731-y PMC6480793

[15] White IR Carpenter J Evans S, et al. Eliciting and using expert opinions about dropout bias in randomized controlled trials. Clin Trials 2007; 4: 125–139. 17456512 10.1177/1740774507077849

[16] Lambert PC Sutton AJ Burton PR, et al. How vague is vague? A simulation study of the impact of the use of vague prior distributions in MCMC using WinBUGS. Stat Med 2005; 24: 2401–2428. 16015676 10.1002/sim.2112

[17] Stevens JW. A note on dealing with missing standard errors in meta-analyses of continuous outcome measures in WinBUGS. Pharm Stat 2011; 10: 374–378. 21394888 10.1002/pst.491

[18] Higgins JPT Whitehead A. Borrowing strength from external trials in a meta-analysis. Stat Med 1996; 15: 2733–2749. 8981683 10.1002/(SICI)1097-0258(19961230)15:24<2733::AID-SIM562>3.0.CO;2-0

[19] Lu G Ades AE. Assessing evidence inconsistency in mixed treatment comparisons. J Am Stat Assoc 2006; 101: 447–459.

[20] Salanti G Ades AE Ioannidis JP. Graphical methods and numerical summaries for presenting results from multiple-treatment meta-analysis: an overview and tutorial. J Clin Epidemiol 2011; 64: 163–171. 20688472 10.1016/j.jclinepi.2010.03.016

[21] Dias S Sutton AJ Ades AE, et al. Evidence synthesis for decision making 2: a generalized linear modeling framework for pairwise and network meta-analysis of randomized controlled trials. Med Decis Making 2013; 33: 607–617. 23104435 10.1177/0272989X12458724 PMC3704203

[22] Gelman A Rubin DB. Inference from iterative simulation using multiple sequences. Stat Sci 1992; 7: 457–472.

[23] Su Y Yajima M. R2jags: Using R to Run ‘JAGS’. Version 0.5-7, https://cran.r-project.org/package=R2jags (2015, 2 December 2020).

[24] R Core Team. R: A language and environment for statistical computing, https://www.r-project.org (2019, 2 December 2020).

[25] Wickham H. Ggplot2: Elegant graphics for data analysis. New York, NY: Springer-Verlag, 2009.

[26] Higgins JP White IR Wood AM. Imputation methods for missing outcome data in meta-analysis of clinical trials. Clin Trials 2008; 5: 225–239. 18559412 10.1177/1740774508091600 PMC2602608

[27] Kemmler G Hummer M Widschwendter C, et al. Dropout rates in placebo-controlled and active-control clinical trials of antipsychotic drugs: a meta-analysis. Arch Gen Psychiatry 2005; 62: 1305–1312. 16330718 10.1001/archpsyc.62.12.1305

[28] Spineli LM Leucht S Cipriani A, et al. The impact of trial characteristics on premature discontinuation of antipsychotics in schizophrenia. Eur Neuropsychopharmacol 2013; 23: 1010–1016. 23639793 10.1016/j.euroneuro.2013.03.006

[29] Spineli LM Kalyvas C Pateras K. Participants' outcomes gone missing within a network of interventions: Bayesian modeling strategies. Stat Med 2019; 38: 3861–3879. 31134664 10.1002/sim.8207 PMC7754380

[30] Spineli LM. Modeling missing binary outcome data while preserving transitivity assumption yielded more credible network meta-analysis results. J Clin Epidemiol 2019; 105: 19–26. 30223064 10.1016/j.jclinepi.2018.09.002

[31] Deeks JJ. Issues in the selection of a summary statistic for meta-analysis of clinical trials with binary outcomes. Stat Med 2002; 21: 1575–1600. 12111921 10.1002/sim.1188

[32] Cipriani A Barbui C Salanti G, et al. Comparative efficacy and acceptability of antimanic drugs in acute mania: a multiple-treatments meta-analysis. Lancet 2011; 378: 1306–1315. 21851976 10.1016/S0140-6736(11)60873-8

[33] Friedrich JO Adhikari NK Beyene J. Ratio of geometric means to analyze continuous outcomes in meta-analysis: comparison to mean differences and ratio of arithmetic means using empiric data and simulation. Stat Med 2012; 31: 1857–1886. 22438170 10.1002/sim.4501

[34] Rhodes KM Turner RM Higgins JP. Empirical evidence about inconsistency among studies in a pair-wise meta-analysis. Res Synth Meth 2016; 7: 346–370. 10.1002/jrsm.1193 PMC5217093 26679486

[35] Higgins JPT, Li T and Deeks JJ (eds). Chapter 6: Choosing effect measures and computing estimates of effect. In: Higgins JPT, Thomas J, Chandler J, et al. (eds) *Cochrane handbook for systematic reviews of interventions*. 6th ed. Cochrane, www.training.cochrane.org/handbook (2019).

[36] Elliott JH Synnot A Turner T, et al. Living systematic review: 1. Introduction-the why, what, when, and how. J Clin Epidemiol 2017; 91: 23–30. 28912002 10.1016/j.jclinepi.2017.08.010

[37] Hughes S Harris J Flack N, et al. The statistician's role in the prevention of missing data. Pharm Stat 2012; 11: 410–416. 22807372 10.1002/pst.1528

[38] Spineli LM. Missing binary data extraction challenges from Cochrane reviews in mental health and Campbell reviews with implications for empirical research. Res Synth Meth 2017; 8: 514–525. 10.1002/jrsm.1268 28961395

[39] Jackson D White IR. When should meta-analysis avoid making hidden normality assumptions? Biom J 2018; 60: 1040–1058. 30062789 10.1002/bimj.201800071 PMC6282623

