# Supplementary material for: Continuous(ly) missing outcome data in network meta-analysis: A one-stage pattern-mixture model approach
Source: Stat Methods Med Res. 2021 Apr;30(4):958–75. doi: 10.1177/0962280220983544 (PMC8209314; doi:10.1177/0962280220983544)
Supplement: sj-pdf-1-smm-10.1177_0962280220983544 - Supplemental material for Continuous(ly) missing outcome data in network meta-analysis: A one-stage pattern-mixture model approach [file sj-pdf-1-smm-10.1177_0962280220983544.pdf]

Supplementary Tables

Table S1. Distribution of percentage of missing outcome data (%MOD) per network

ID	minimum	1st quartile	median	mean	3rd quartile	maximum
			Total %MOD			
1	0.00	0.00	0.00	1.02	1.39	4.37
2	0.00	0.66	5.88	15.55	33.33	55.84
3	0.00	0.67	1.85	6.48	5.19	49.89
4	0.00	0.00	0.32	4.46	6.54	18.84
5	0.00	0.00	1.09	6.83	10.63	30.77
			The difference in %MOD between compared arms[1]			
1	0.00	0.00	0.00	0.12	0.03	0.79
2	0.00	0.04	1.69	13.01	7.32	66.39
3	0.00	0.75	1.69	6.99	3.97	66.33
4	0.00	0.00	0.19	2.17	2.68	10.95
5	0.00	0.00	0.00	1.89	3.41	8.96

[1]In multi-arm trials, the difference between the arm with the maximum and the arm with the minimum %MOD is calculated.

References of collected networks with a primary continuous outcome

1. Wandel S, Jüni P, Tendal B, Nüesch E, Villiger PM, Welton NJ, Reichenbach S, Trelle S. Effects of glucosamine, chondroitin, or placebo in patients with osteoarthritis of hip or knee: network meta-analysis. *BMJ* 2010;341:c4675.

2. Stowe R, Ives N, Clarke CE, Handley K, Furmston A, Deane K, van Hilten JJ, Wheatley K, Gray R. Meta-analysis of the comparative efficacy and safety of adjuvant treatment to levodopa in later Parkinson's disease. *Mov Disord* 2011;26(4):587-98.

3. Cipriani A, Barbui C, Salanti G, Rendell J, Brown R, Stockton S, Purgato M, Spineli LM, Goodwin GM, Geddes JR. Comparative efficacy and acceptability of antimanic drugs in acute mania: a multiple-treatments meta-analysis. *Lancet* 2011;378(9799):1306-15.

4. Squires H, Simpson E, Meng Y, Harnan S, Stevens J, Wong R, Thomas S, Michaels J, Stansby G. A systematic review and economic evaluation of cilostazol, naftidrofuryl oxalate, pentoxifylline and inositol nicotinate for the treatment of intermittent claudication in people with peripheral arterial disease. *Health Technol Assess* 2011;15(40):1-210.

5. Schwingshackl L, Missbach B, Dias S, König J, Hoffmann G. Impact of different training modalities on glycaemic control and blood lipids in patients with type 2 diabetes: a systematic review and network meta-analysis. *Diabetologia* 2014;57(9):1789-97.

Table S2. Posterior mean and 95% CrI of IMRoM under different structural assumptions

Network	Interventions	Assumptions about the structure of log IMRoM (ω_{ik})			
		**Common-within-network**		**Intervention-specific**	
		**Hierarchical** $\omega_{ik} \sim N(\Delta, \sigma^2)$, $\Delta \sim N(0, 0.2^2)$, $\sigma \sim U(0,0.2)$	**Identical** $\omega_{ik} = \omega$, $\omega \sim N(0, 0.2^2)$	**Hierarchical** $\omega_{ik} \sim N\left(\Delta_{t_{ik}}, \sigma^2_{t_{ik}}\right)$, $\Delta_{t_{ik}} \sim N(0, 0.2^2)$, $\sigma_{t_{ik}} \sim U(0,0.2)$	**Identical** $\omega_{ik} = \omega_{t_{ik}}$, $\omega_{t_{ik}} \sim N(0, 0.2^2)$
Antiparkinsonian drugs [2]	COMTI+LD	-0.01 (-0.37, 0.36)	-0.02 (-0.39, 0.36)	-0.11 (-0.47, 0.27)	-0.11 (-0.48, 0.27)
	DA+LD			0.03 (-0.36, 0.42)	0.02 (-0.36, 0.41)
	MAOBI +LD			0.01 (-0.38, 0.40)	0.02 (-0.37, 0.40)
	PBO+LD			0.07 (-0.35, 0.46)	0.06 (-0.34, 0.45)
Training modalities [5]	Aerobic	0.05 (-0.36, 0.46)	0.06 (-0.34, 0.46)	0.06 (-0.33, 0.45)	0.06 (-0.34, 0.45)
	Combined training			-0.02 (-0.40, 0.36)	-0.03 (-0.42, 0.35)
	Resistance			0.03 (-0.36, 0.43)	0.03 (-0.36, 0.43)

COMTI+LD, catechol-O-methyl transferase inhibitors plus levodopa; CrI, credible interval; DA+LD, dopamine agonist plus levodopa; IMRoM, informative missingness ratio of means; MAOBI+LD, monoamine oxidase type B inhibitors plus levodopa, PBO+LD, placebo plus levodopa.

Table S3. Posterior mean and 95% CrI of IMDoM under different structural assumptions†

SENSITIVITY ANALYSIS					
		Assumptions about the structure of IMDoM (φ_{ik})			
		Common-within-network		Intervention-specific	
**Network**	**Interventions**	**Hierarchical** $\varphi_{ik} \sim N(\Delta, \sigma^2)$, $\Delta \sim N(0, 3^2)$, $\sigma \sim U(0,3)$	**Identical** $\varphi_{ik} = \varphi$, $\varphi \sim N(0, 3^2)$	**Hierarchical** $\varphi_{ik} \sim N\left(\Delta_{t_{ik}}, \sigma^2_{t_{ik}}\right)$, $\Delta_{t_{ik}} \sim N(0, 3^2)$, $\sigma_{t_{ik}} \sim U(0,3)$	**Identical** $\varphi_{ik} = \varphi_{t_{ik}}$, $\varphi_{t_{ik}} \sim N(0, 3^2)$
Antiparkinsonian drugs[12]	COMTI+LD	1.17 (0.15, 2.17)	1.20 (0.34, 2.07)	1.69 (0.32, 3.02)	1.69 (0.61, 2.75)
	DA+LD			-0.11 (-3.10, 2.57)	0.22 (-1.79, 2.24)
	MAOBI +LD			0.18 (-3.17, 3.46)	0.20 (-1.73, 2.11)
	PBO+LD			1.18 (-1.40, 3.79)	1.27 (-0.67, 3.25)
Training modalities[13]	Aerobic	-0.29 (-3.65, 3.20)	-0.54 (-3.86, 3.07)	-0.24 (-3.11, 2.68)	-0.51 (-3.09, 2.25)
	Combined training			1.45 (-2.33, 5.21)	1.62 (-1.85, 5.13)
	Resistance			-1.01 (-4.23, 2.18)	-1.23 (-4.21, 1.82)

COMTI+LD, catechol-O-methyl transferase inhibitors plus levodopa; CrI, credible interval; DA+LD, dopamine agonist plus levodopa; IMDoM, informative missingness difference of means; MAOBI+LD, monoamine oxidase type B inhibitors plus levodopa, PBO+LD, placebo plus levodopa.

†Mean difference with informative missingness difference of means.

Table S4. Posterior mean and 95% CrI of IMRoM under different structural assumptions

SENSITIVITY ANALYSIS					
		Assumptions about the structure of log IMRoM (ω_{ik})			
		Common-within-network		Intervention-specific	
**Network**	**Interventions**	**Hierarchical** $\omega_{ik} \sim N(\Delta, \sigma^2)$, $\Delta \sim N(0, 0.4^2)$, $\sigma \sim U(0,0.4)$	**Identical** $\omega_{ik} = \omega$, $\omega \sim N(0, 0.4^2)$	**Hierarchical** $\omega_{ik} \sim N\left(\Delta_{t_{ik}}, \sigma^2_{t_{ik}}\right)$, $\Delta_{t_{ik}} \sim N(0, 0.4^2)$, $\sigma_{t_{ik}} \sim U(0,0.4)$	**Identical** $\omega_{ik} = \omega_{t_{ik}}$, $\omega_{t_{ik}} \sim N(0, 0.4^2$
Antiparkinsonian drugs[12]	COMTI+LD	-0.04 (-0.76, 0.72)	-0.05 (-0.74, 0.65)	-0.32 (-1.00, 0.36)	-0.33 (-1.01, 0.34)
	DA+LD			0.09 (-0.67, 0.81)	0.08 (-0.67, 0.80)
	MAOBI +LD			0.04 (-0.73, 0.78)	0.06 (-0.71, 0.80)
	PBO+LD			0.24 (-0.62, 1.07)	0.25 (-0.58, 1.08)
Training modalities[13]	Aerobic	0.29 (-0.58, 1.18)	0.29 (-0.57, 1.16)	0.24 (-0.59, 1.04)	0.24 (-0.58, 1.05)
	Combined training			-0.10 (-0.84, 0.64)	-0.10 (-0.85, 0.63)
	Resistance			0.14 (-0.68, 0.95)	0.14 (-0.67, 0.95)

COMTI+LD, catechol-O-methyl transferase inhibitors plus levodopa; CrI, credible interval; DA+LD, dopamine agonist plus levodopa; IMDoM, informative missingness difference of means; MAOBI+LD, monoamine oxidase type B inhibitors plus levodopa, PBO+LD, placebo plus levodopa.

Table S5. The posterior mean of residual deviance for all one-stage pattern-mixture models per network

ID	Number of points	Exclusion	Common-within-network		Trial-specific		Intervention-specific		Correlated	Uncorrelated
			Identical	Hierarchical	Identical	Hierarchical	Identical	Hierarchical		
Mean Difference										
1	14	12.63	12.61	12.63	12.60	12.60	12.53	12.57	12.62	12.64
2	58	**59.42**	57.27	57.39	58.46	58.36	57.11	57.49	**58.64**	**58.88**
3	141	**156.63**	**157.12**	**157.12**	**156.89**	**156.97**	**156.82**	**157.02**	**156.94**	**156.96**
4	17	16.98	16.95	17.06	16.94	17.02	17.14	17.16	17.07	17.11
5	33	32.39	32.22	31.79	32.17	31.60	31.85	31.29	31.50	31.22
Standardised Mean Difference										
1	14	12.70	12.67	12.66	12.67	12.64	12.61	12.60	12.68	12.72
2	58	**61.56**	56.88	56.87	58.02	57.98	56.58	56.96	58.38	58.53
3	141	**158.03**	**158.52**	**158.58**	**158.15**	**158.02**	**158.17**	**158.20**	**158.00**	**158.09**
4	17	17.02	16.99	16.76	16.90	16.69	17.08	16.84	16.68	16.46
5	33	32.06	31.96	31.95	31.99	31.96	31.90	31.41	32.04	31.87
Ratio of Means (in logarithmic scale)										
1	14	12.10	12.12	12.12	12.10	12.07	12.10	12.11	12.13	12.13
2	58	**63.49**	**63.21**	**63.13**	**63.18**	**63.24**	**63.33**	**63.32**	**63.18**	**63.31**
3	141	140.97	140.87	140.78	140.95	141.05	141.03	140.85	140.90	140.80
4	17	17.24	17.14	17.24	17.21	17.23	17.22	17.16	17.23	17.22
5	33	29.26	29.32	29.32	29.31	29.35	29.37	29.34	29.37	29.33

Models with lack of fit (posterior mean of residual deviance is greater than the number of observed independent points) are indicated in bold.
The references of the networks can be found after Table S1.

Supplementary Figures

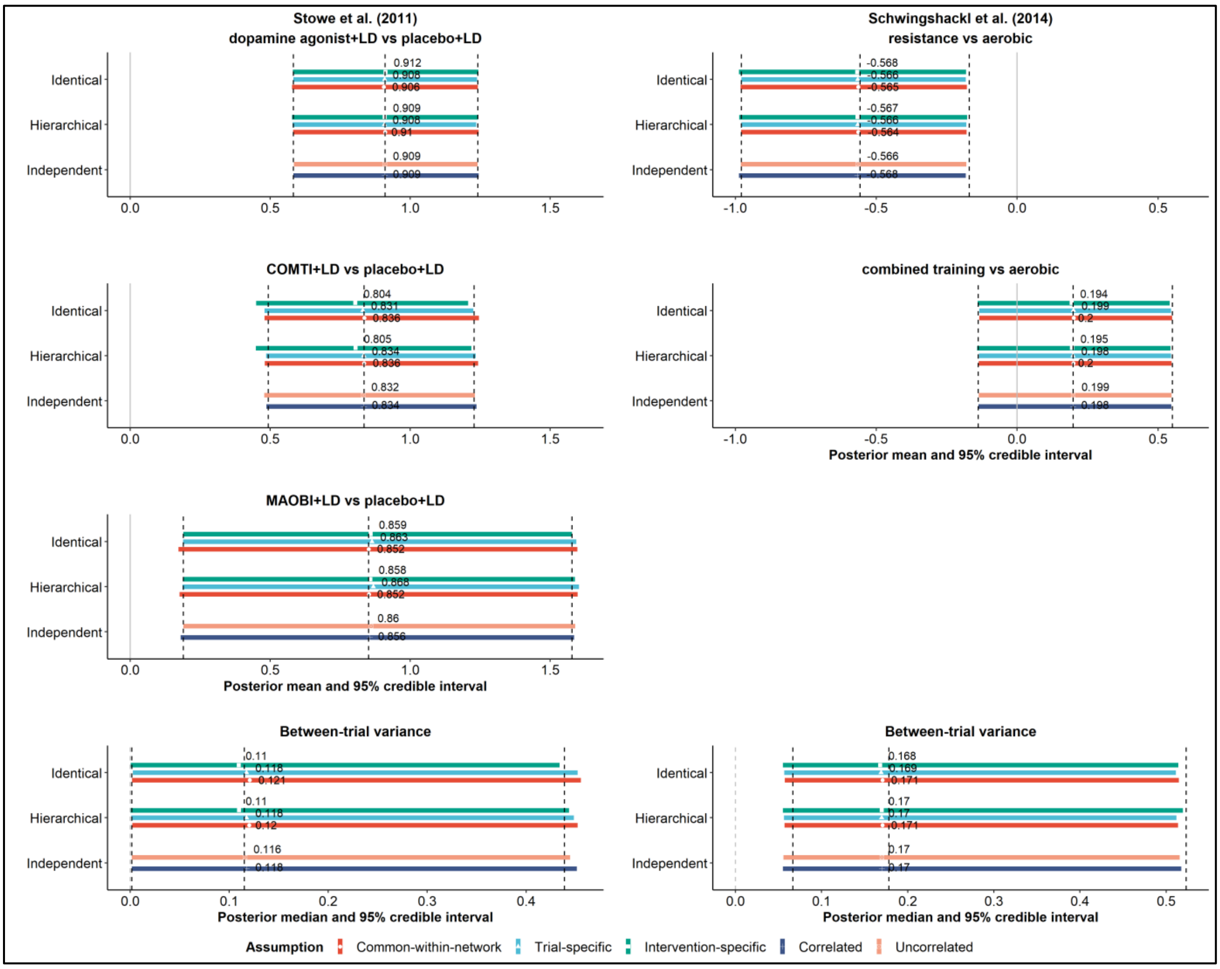

Figure S1. Interval plots for the ratio of means in the logarithmic scale for comparisons with the reference intervention of each network, and the common between-trial variance using the network of Stowe et al. (left panel) and the network of Schwingshackl et al. (right panel). The one-stage pattern-mixture model under the hierarchical, identical, and independent structure of informative missingness ratio of means in the logarithmic scale (log IMRoM) for the assumption of common-within-network, trial-specific, intervention-specific, within-trial correlated and uncorrelated log IMRoMs: The vertical dashed lines refer to the point estimate and 95% credible interval under the available case analysis. COMTI+LD, catechol-O-methyl transferase inhibitors plus levodopa; MAOBI+LD, monoamine oxidase type B inhibitors plus levodopa.

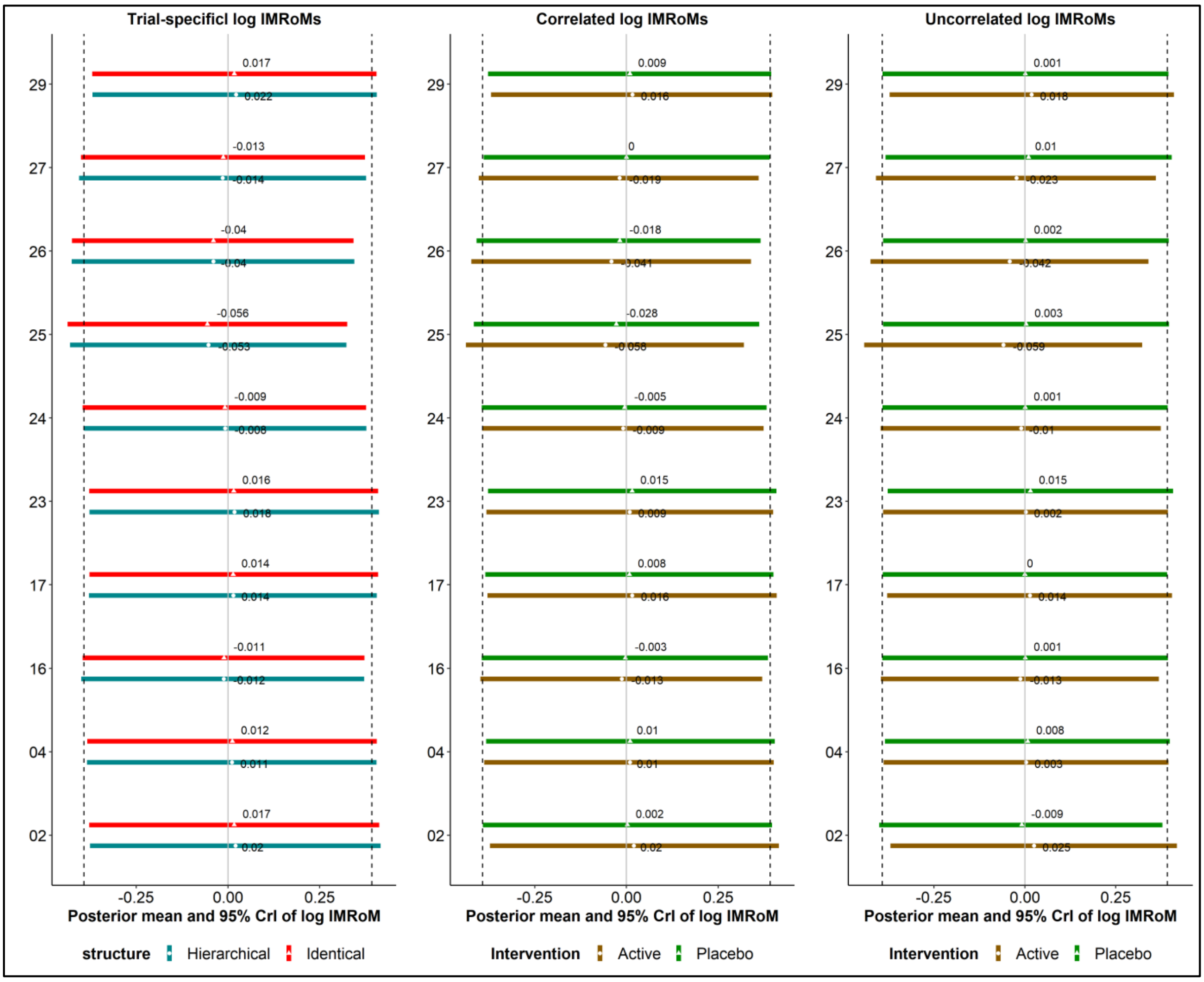

Figure S2. Interval plots on the posterior distribution of the informative missingness ratio of means in the logarithmic scale (log IMRoM) using the ratio of means in the logarithmic scale in the network of Stowe et al. the one-stage pattern-mixture model under the hierarchical and identical structure assuming trial-specific log IMRoMs and under the independent structure assuming within-trial correlated and uncorrelated log IMRoMs. The vertical lines indicate the prior distribution for log IMRoM. COMTI+LD, catechol-O-methyl transferase inhibitors plus levodopa; CrI, credible interval; DA+LD, dopamine agonist plus levodopa; MAOBI+LD, monoamine oxidase type B inhibitors plus levodopa, PBO+LD, placebo plus levodopa.

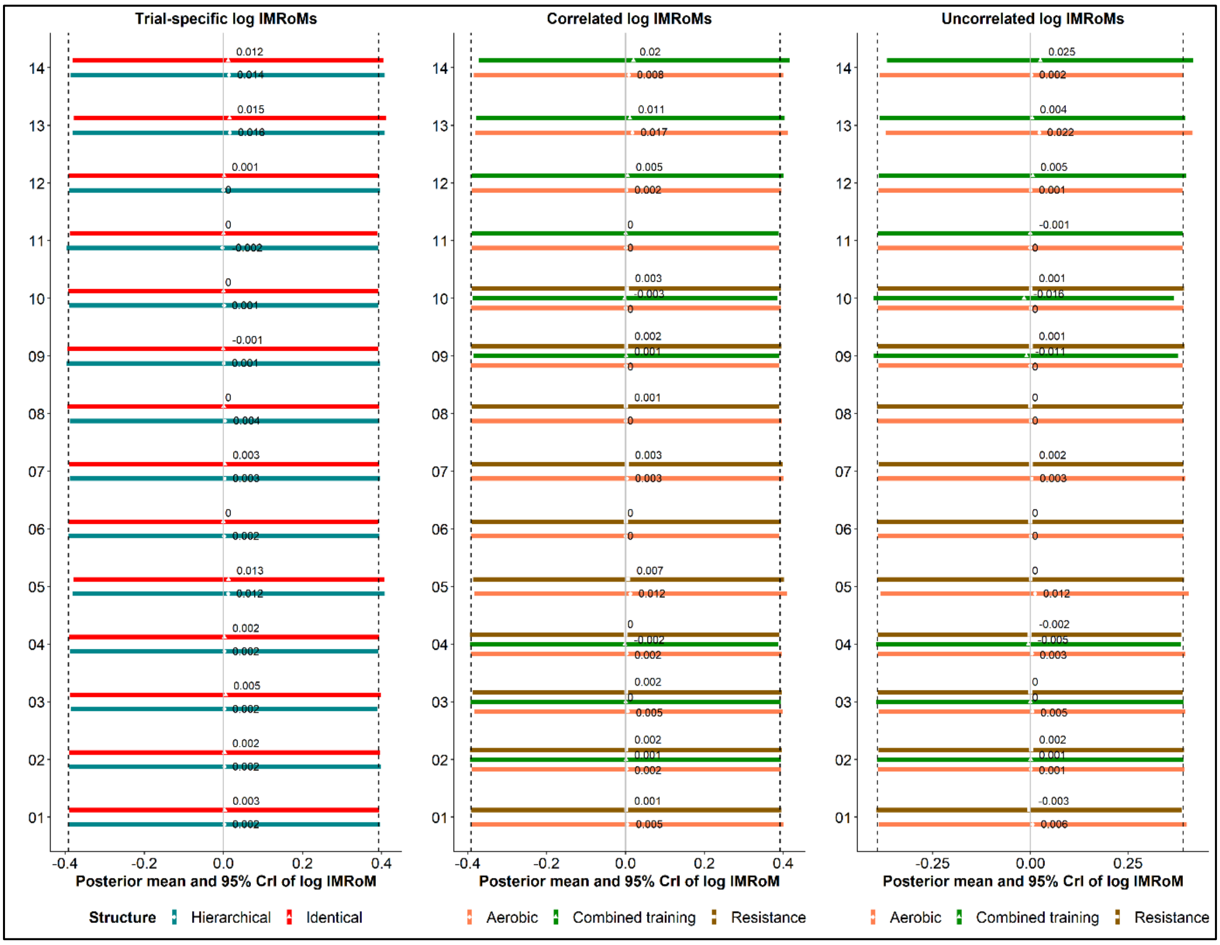

Figure S3. Interval plots on the posterior distribution of the informative missingness ratio of means in the logarithmic scale (log IMRoM) using the ratio of means in the logarithmic scale in the network of Schwingshackl et al. The one-stage pattern-mixture model under the hierarchical and identical structure assuming trial-specific log IMRoMs and under the independent structure assuming within-trial correlated and uncorrelated log IMRoMs. The vertical lines indicate the prior distribution for log IMRoM. CrI, credible interval.

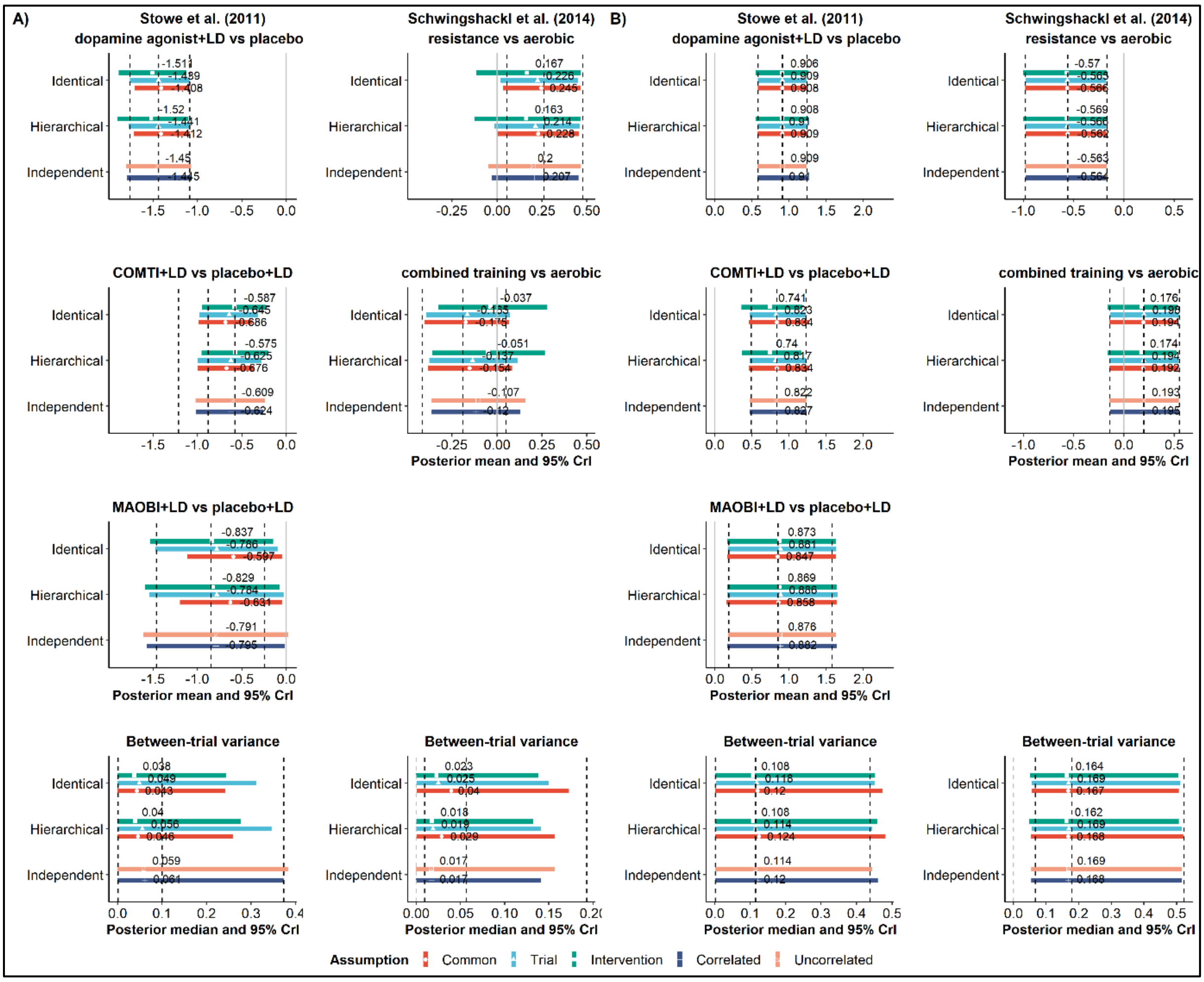

Figure S4. Interval plots for the mean difference (panel A)) and the ratio of means in the logarithmic scale (panel B)) for comparisons with the reference intervention of each network, and the common between-trial variance for the network of Stowe et al. and the network of Schwingshackl et al. The one-stage pattern-mixture model under the hierarchical, identical, and independent structure of the informative missingness difference of means (IMDoM) for mean difference, and the informative missingness ratio of means in the logarithmic scale (log IMRoM) for log RoM under the assumption of common-within-network, trial-specific, intervention-specific, within-trial correlated and uncorrelated log IMRoMs. Use of prior variance equal to 3^2 for IMDoM and equal to 0.4^2 for log IMRoM. The vertical dotted lines refer to the point estimate and 95% CrI under the available case analysis. COMTI+LD, catechol-O-methyl transferase inhibitors plus levodopa; CrI, credible interval; MAOBI+LD, monoamine oxidase type B inhibitors plus levodopa.

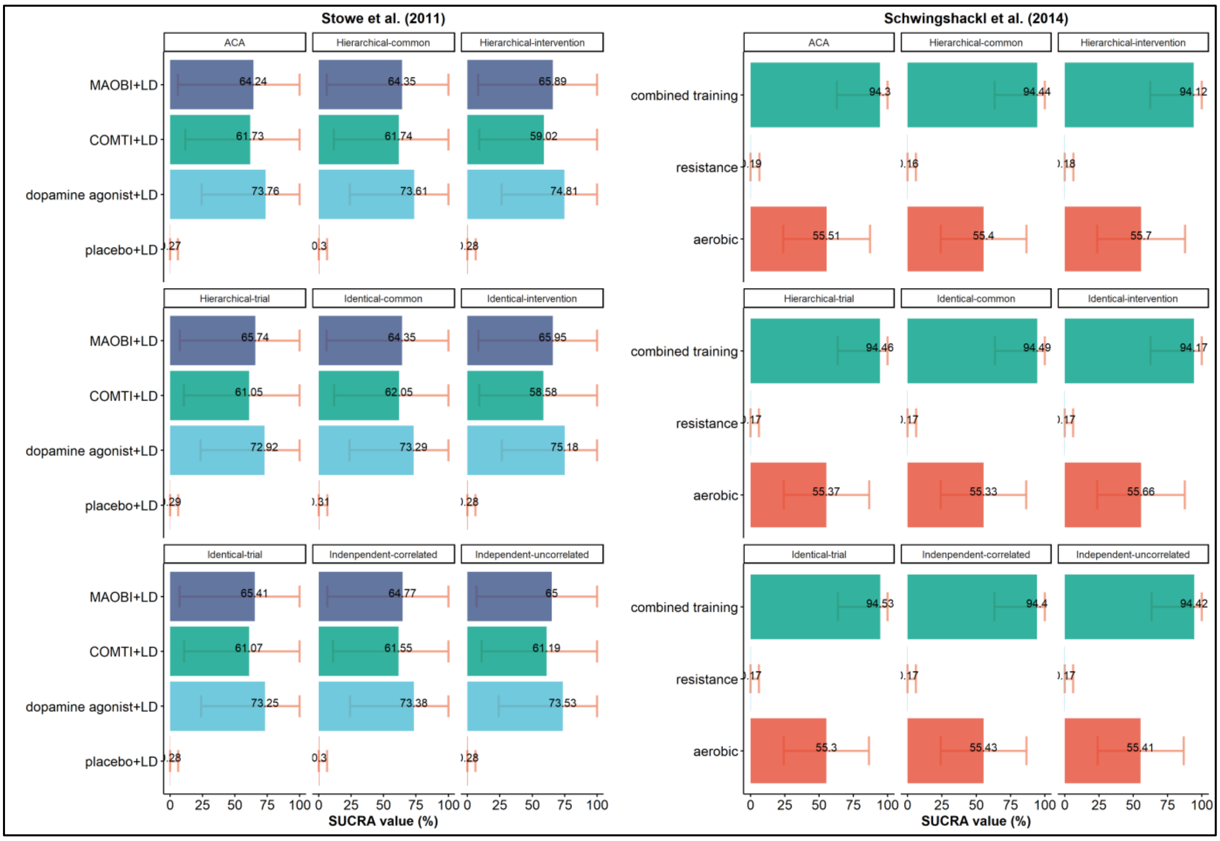

Figure S5. Barplots on the SUCRA values under the ratio of means in the logarithmic scale (log RoM) for the network of Stowe et al. (left panel) and Schwingshackl et al. (right panel). Application of the one-stage pattern-mixture model under the hierarchical, identical, and independent structure of informative missingness ratio of means in the logarithmic scale (log IMRoM) under the common-within-network, trial-specific, intervention-specific, within-trial correlated and uncorrelated assumptions. Use of prior variance equal to 0.2^2 for log IMRoM. Results under the ACA are also presented. ACA, available case analysis; COMTI+LD, catechol-O-methyl transferase inhibitors plus levodopa; MAOBI+LD, monoamine oxidase type B inhibitors plus levodopa; SUCRA, surface under the cumulative ranking.
